# Time Clustered Sampling Can Inflate the Inferred Substitution Rate in Foot-And-Mouth Disease Virus Analyses

**DOI:** 10.1371/journal.pone.0143605

**Published:** 2015-12-02

**Authors:** Casper-Emil T. Pedersen, Peter Frandsen, Sabenzia N. Wekesa, Rasmus Heller, Abraham K. Sangula, Jemma Wadsworth, Nick J. Knowles, Vincent B. Muwanika, Hans R. Siegismund

**Affiliations:** 1 Department of Biology, University of Copenhagen, Copenhagen, Denmark; 2 Foot-and-Mouth Disease Laboratory, Embakasi, Nairobi, Kenya; 3 The Pirbright Institute, Ash Road, Pirbright, United Kingdom; 4 Department of Environmental Management, College of Agricultural and Environmental Sciences, Makerere University, Kampala, Uganda; University of Sydney, AUSTRALIA

## Abstract

With the emergence of analytical software for the inference of viral evolution, a number of studies have focused on estimating important parameters such as the substitution rate and the time to the most recent common ancestor (*t*_MRCA_) for rapidly evolving viruses. Coupled with an increasing abundance of sequence data sampled under widely different schemes, an effort to keep results consistent and comparable is needed. This study emphasizes commonly disregarded problems in the inference of evolutionary rates in viral sequence data when sampling is unevenly distributed on a temporal scale through a study of the foot-and-mouth (FMD) disease virus serotypes SAT 1 and SAT 2. Our study shows that clustered temporal sampling in phylogenetic analyses of FMD viruses will strongly bias the inferences of substitution rates and *t*_MRCA_ because the inferred rates in such data sets reflect a rate closer to the mutation rate rather than the substitution rate. Estimating evolutionary parameters from viral sequences should be performed with due consideration of the differences in short-term and longer-term evolutionary processes occurring within sets of temporally sampled viruses, and studies should carefully consider how samples are combined.

## Introduction

Foot-and-mouth disease (FMD) is a highly contagious vesicular disease that occurs in cloven-hoofed livestock and wildlife animals [1]. The causative agent, FMD virus (FMDV), is a small non-enveloped virus with a positive sense single-stranded RNA genome approximately 8.4kb long. It belongs to the genus *Aphthovirus* within the family *Picornaviridae* [2]. The viral genome encodes a polyprotein, which is cleaved and processed into several structural and non-structural proteins [2]. The FMDV polyprotein produces 15 different mature proteins, where VP1–3 constitute the structural proteins that are responsible for the surface of the capsid. The VP1 contains important epitopes that are recognized by neutralizing antibodies generated by the mammalian immune system [2] and also the integrin receptor binding motif. In consequence of these constraints, the VP1 coding sequence has been found to be under both positive and negative selection [3]. Additionally, the VP1 coding region has been used extensively to study the evolutionary relationship within serotypes, including the inference of substitution rates and time to the most recent common ancestor (*t*_MRCA_) [4–8].

FMD viruses are widely distributed and are divided into seven serotypes (SAT 1, SAT 2, SAT 3, O, A, C, and Asia 1) [8] with some of these being further differentiated into topotypes [9]. The SAT serotypes are generally confined to sub-Saharan Africa [10], but SAT 2 has recently been found in North Africa and the Middle East [11], whereas serotypes O, A, and Asia 1 are found on a larger geographical scale. Serotype C was most recently been recorded in Kenya and from an outbreak in Brazil, both in 2004 [12,13], and may now be extinct. Many rapidly evolving viruses occur in outbreaks interspersed with periods where they occur at lower frequency, which clearly shape the genealogies and genetic diversity patterns of viruses sampled over time [14,15]. Duchêne *et al*. [16] and Ho *et al*. [17] found a strong negative relationship between estimates of substitution rates and the evolutionary time scale for major groups of viruses. They relate this observation to the combined effect of site saturation and purifying selection and argue that substitution rates must be considered as a dynamic property of molecular evolution.

Studies by Duffy *et al*. [18] and Sangula *et al*. [3] stressed the potential biases caused by opportunistic sampling schemes in regards to FMDV; whenever multiple samples are collected when the viral population is largest (during an outbreak), the inferred rates will reflect a value more akin to the mutation rate than the substitution rate. During outbreaks, high levels of polymorphisms are generated due to the combination of an explosive growth phase and the error-prone RNA dependent RNA polymerase [18]. The majority of these polymorphisms are lost over time and do not contribute to fixed substitutions in the subsequent virus lineages. This leads to variable levels and patterns of polymorphisms as temporal sampling distance increases. Consequently, evolutionary analyses investigating temporally sampled sequences, including densely sampled sequence data, can result in a biased estimate of the substitution rate [18]. Inference of the long-term substitution rate and correspondingly the *t*_MRCA_ are likely to be affected by a failure to recognize this variability, and this is problematic since these parameters are crucial for tracking transmission events through evolutionary analyses [19] and for obtaining a more complete understanding of RNA virus dynamics in general.

In this study, we assessed the problems associated with the temporal structure of sampling in FMD virus studies with a particular focus on sequences obtained from Africa. We accomplish this by inferring the substitution rates and *t*_MRCA_ in samples from two different serotypes of FMD virus sampled either in a temporally clustered fashion or more uniformly. Using this approach, we tested the hypothesis that FMDV sequence data sets including densely sampled sequences can bias overall estimates of evolutionary rates and associated parameters such as the time to the most recent common ancestor.

## Materials and Methods

### Virus isolates

The FMDV serotypes investigated in this study were a combination of sequences retrieved from GenBank (see accession numbers in S1 Appendix) and sequences provided by the FAO World Reference Laboratory for FMD. SAT 1 sequences included in this study had been sampled between 1937 and 2010 and include two densely sampled outbreaks, one from Niger-Nigeria in 1975–1976 and a more recent outbreak in Kenya 2010 (both outbreaks are indicated by an asterisk in S1 Appendix). SAT 2 isolates had been sampled between 1948 and 2012 and include four densely sampled outbreaks: South African sequences from 2001, Kenyan sequences from 2007, Ethiopian sequences from 2009 and sequences from Egypt sampled in 2012 (indicated by an asterisk in S1 Appendix). Input files for BEAST v. 1.8.2 [20] (http://beast.bio.ed.ac.uk/) are produced using the BEAUti package. These files contain the settings for each data set and are in the XML format. All individual XML files used for this study can be seen in S2 Appendix.

### Definition of temporal sample clusters

**Fig 1:**
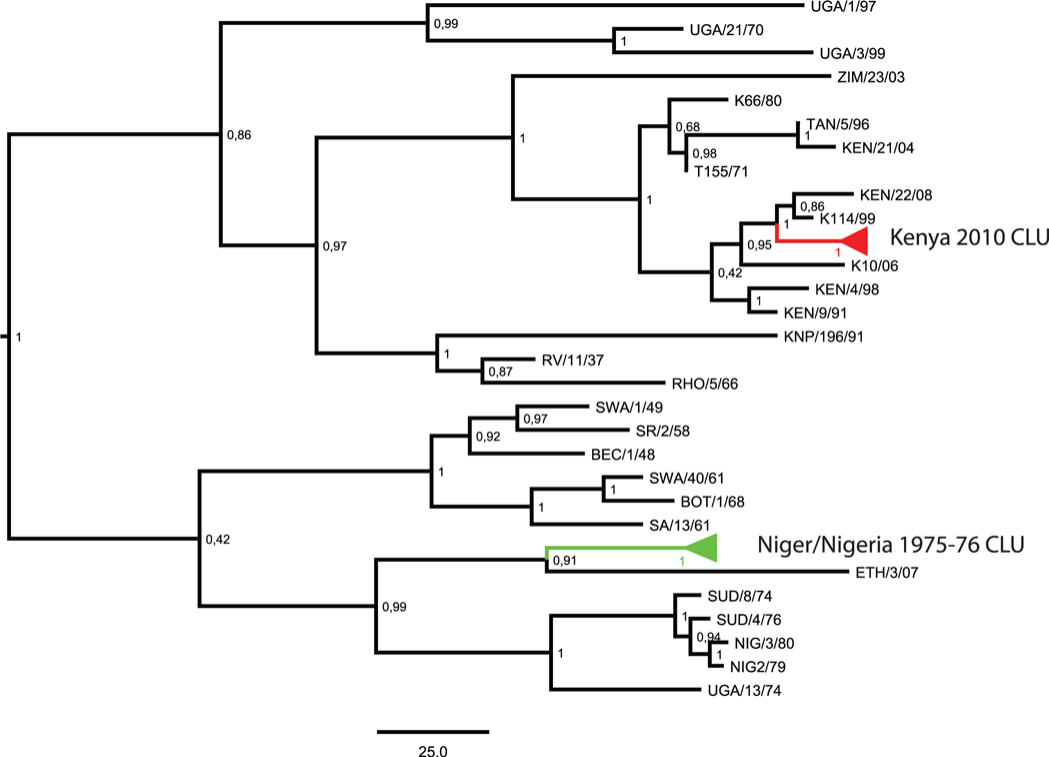
SAT 1 Phylogenetic Tree. Markov Chain Monte Carlo phylogenetic tree generated using the combined SAT 1 data set including both chronologically sampled (CHR) data sets and temporal sample clusters (CLU). Temporal sample clusters are collapsed and coloured. Posterior probabilities are given for each node and the scale bar indicates a branch length corresponding to 25 years.

**Fig 2:**
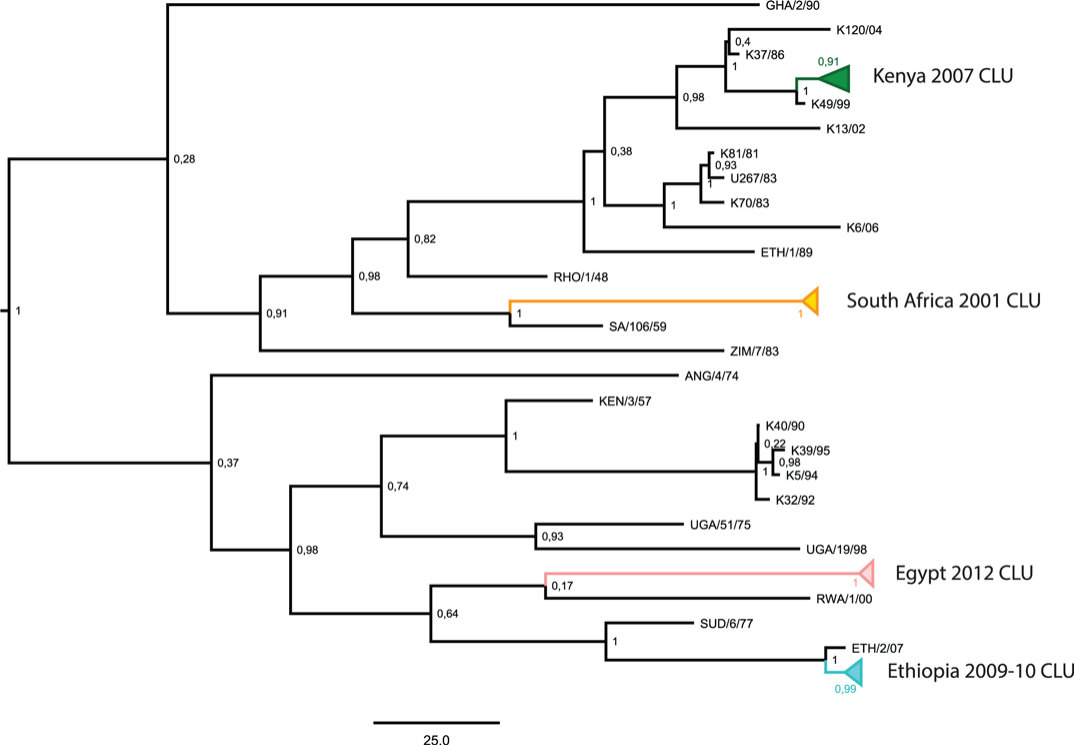
SAT 2 Phylogenetic Tree. Markov Chain Monte Carlo phylogenetic tree generated using the combined SAT 2 data set including both chronologically sampled (CHR) data sets and temporal sample clusters (CLU). Temporal sample clusters are collapsed and coloured. Posterior probabilities are given for each node and the scale bar indicates a branch length corresponding to 25 years.

An FMD outbreak is defined by The World Organization for Animal Health (OIE) as: “One or more cases (individuals infected) found within an epidemiological unit (a group of animals which share the same risk of pathogen exposure)” [21]. We defined temporal sample clusters (CLU) as groups of samples that fulfilled the following three requirements: they were i) sampled in relatively close geographical proximity (within the same or neighbouring countries) ii) sampled within 18 months of time and iii) belonged to the same topotype [9]. Samples that did not fulfil these requirements were assigned to chronologically sampled (CHR) data sets. Exceptions were samples collected less than 18 months apart that belonged to different topotypes, which were instead included in the CHR data sets by randomly choosing a sample from each topotype within the 18 month window. In addition, we analysed combined data sets for both serotypes that included both the temporal sample clusters and the chronologically sampled sequences. The overall phylogenetic divergence and tree topology of the two serotypes, including the CLU sequences, can be seen in Figs 1 and 2. The settings used to construct these trees can be seen in the next section. Furthermore, we found two cases of identical DNA sequences from different sampling sites (i.e. geographical origin) and sampling years. These samples were collected 7 years apart. Considering the high substitution rate in FMDV it is extremely unlikely to have identical FMD isolates circulating over such a time span. Consequently, we assumed these to represent cases of contamination or mislabelling and the samples were removed.

### Phylogenetic analyses

Sequences were aligned using the ClustalW-algorithm [22] implemented in Geneious version 6.1.6 (http://www.geneious.com/). JModelTest version 2.1.3 [23] was used to determine the best fitting nucleotide substitution model. Twenty four different substitution models were compared through a hierarchical likelihood ratio test using this tool, and all were evaluated by using both Bayesian Information Criterion (BIC) and Akaike Information Criterion (AIC) which produced identical model choices. The GTR+I+Γ [24] was selected for the SAT 1 and SAT 2 CHR and combined data sets. The HKY+I+Γ model [25] was selected for the SAT 1 and SAT 2 CLU data sets. We constructed the maximum clade credibility trees using TreeAnnotator (http://beast.bio.ed.ac.uk/treeannotator) discarding the first 10% of sampled trees as burn-in. These trees were visualized using FigTree version 1.4.2 (http://beast.bio.ed.ac.uk/figtree).

Substitution rates and phylogenetic tree depths were estimated using a Bayesian statistical approach implemented in BEAST v. 1.8.2 using the selected substitution models. The method exploits temporally sampled data with known sampling times to infer substitution rates along lineages while co-estimating phylogenetic trees and *t*_MRCA_.

Three demographic models (the constant population size model, the exponential growth population model and the Bayesian skyline coalescent model) were investigated and compared. We chose these models as they are the most commonly used and collectively cover a large span of demographic priors. As the posterior for the coefficient of variation in the relaxed clock models always excluded zero–meaning there was a clear signal of rate heterogeneity–we used a relaxed exponential clock throughout the analyses. We used a UPGMA starting tree and the MCMC chains were run long enough (1 × 10^8^) to obtain Effective Sample Size (ESS) above 200 for all parameters. This approach was used for both the CLU and CHR data sets. The results were assessed with Tracer software v. 1.5 [26] with a 10% burn-in. Statistical uncertainty in the results was presented as the lower 2.5%, the average and the upper 97.5% of the highest probability density (HPD) interval.

We conducted ten date permutations for each data set and investigated these alongside the original data set to test for temporal signal (similar to what was done by Ramsden *et al*. [27]). Specifically, we wanted to see whether the substitution rates obtained from the permuted data sets overlap with the substitution rates inferred for the true data set. We considered that temporal signal was present when the mean estimate (shown by a circle in our plots) from the true data set did not overlap with the 95% HPD intervals of the estimates from the date-permuted data sets [28]. We limited this analysis to the constant and exponential demographic models.

### Detecting selection and recombination

The two serotypes were analysed separately and further subdivided as described above. We assessed *dN/dS* ratios using the single-likelihood ancestor counting (SLAC) method (*P* = 0.1) [29] available within the HyPhy-package and accessed through the Datamonkey webserver (www.datamonkey.org). This likelihood approach estimates the best fitting codon model using fixed substitution rates and branch lengths to infer the global *dN/dS* ratio.

To test for recombination we used the Single Break Point (SBP) method [30] available on the Datamonkey server. We tested both serotypes individually and combined in an all-sequence data set. To add confidence to this analysis, additional methods implemented within RDP 4.22 software [31] were used on all data sets, including Chimaera [32], GENECONV [33] and MaxChi [34].

## Results

### Date permutation

We conducted date permutations for each data set to test for a temporal signal. For SAT 1, we found the strongest signal in the combined and the NIGER/NIGERIA 75/76 data sets while the KENYA 2010 CLU and SAT 1 CHR data sets carried minor or no evidence of temporal signal. These findings were identical across demographic models (See S1–S4 Figs for the exponential demographic model and S11–S14 Figs for the constant demographic model). The results for the SAT 2 serotype showed contrasting results. Using the exponential demographic model, we observed strong support for a temporal signal in all but the Kenya 2007 CLU data set. However, under the constant model, only the combined, Ethiopian 2009 CLU and the CHR data sets showed a temporal signal (see S5–S10 Figs for the exponential model and S15–S20 Figs for the constant model).

### Phylogenetic and rate inferences

The SAT 1 phylogenetic tree shows two main clades. The first clade includes primarily the northern sequences and the KENYA 2010 CLU, while the second clade contains the southern and western sequences including the NIGER/NIGERIA 75/76 CLU. Both of the CLUs are monophyletic with maximum posterior probability (Fig 1). For SAT 2, we observed divergence of two main clades (Fig 2). Estimates from the BEAST analyses of the two serotypes revealed elevated evolutionary rates for the CLU data sets compared to both the CHR and combined data sets in all serotypes (Table 1 **)**. This difference was found under all demographic models. The SAT 1 combined data set yielded median rates of 2.8 (1.7–4.1), 3.0 (2.0–4.3) and 1.5 (0.9–2.1) × 10^−3^ substitutions/nucleotide/year (s/nt/y) for constant, exponential and skyline demographic models, respectively. The median rates for the SAT 1 CHR data set were much smaller, 0.1 (0.00001–0.4), 0.2 (0.01–0.6) and 0.1 (0.0001–0.4) × 10^−3^ s/nt/y. Data sets including only SAT 1 CLU (both either SAT 1 KENYA 2010 or SAT 1 NIGER/NIGERIA 75/76) displayed an even higher rate of evolution than the CHR data set (Table 1 **)**.

**Table 1:** Bayesian estimates of substitution rates and *t*_MRCA_ for the two serotopes.

**SAT1 data sets**			**Substitution rates (s/bp/yr)**	**Substitution rates (s/bp/yr)**	**Substitution rates (s/bp/yr)**	***t***_**MRCA**_ **(y BP)**	***t***_**MRCA**_ **(y BP)**	***t***_**MRCA**_ **(y BP)**
** **	***n***	** Coverage**	**Constant**	**Exponential**	**Skyline**	**Constant**	**Exponential**	**Skyline**
SAT 1 combined	96	1937–2010	2.8 × 10^−3^ (1.7 × 10^−3^–4.1 × 10^−3^)	3.0 × 10^−3^ (2.0 × 10^−3^–4.3 × 10^−3^)	1.5 × 10^−3^ (0.9 × 10^−3^–2.1 × 10^−3^)	223 (121–395)	191(127–278)	386 (233–590)
KENYA 2010 outbreak	56	2010	6.7 × 10^−3^ (3.1 × 10^−3^–10.8 × 10^−3^)	3.7 × 10^−3^ (0.1 × 10^−3^–7.7 × 10^−3^)	5.7 × 10^−3^ (1.5 × 10^−3^–10.2 × 10^−3^)	1.8 (1.0–3.1)	2.1 (1.0–7.5)	1.4 (0.8–3.1)
NIGER/NIGERIA outbreak	12	1975–1977	12.9 × 10^−3^ (3.9 × 10^−3^–27.0 × 10^−3^)	12.9 × 10^−3^ (3.7 × 10^−3^–26.9 × 10^−3^)	9.7 × 10^−3^ (3.4 × 10^−3^–18.3 × 10^−3^)	2.4 (2.0–3.8)	2.4 (2.1–3.7)	2.4 (2.1–3.2)
SAT 1 chronologically sampled	28	1937–2008	0.1 × 10^−3^ (0.00001 × 10^−3^–0.4 × 10^−3^)	0.2 × 10^−3^ (0.01 × 10^−3^–0.6 × 10^−3^)	0.1 × 10^−3^ (0.0001 × 10^−3^–0.4 × 10^−3^)	4738 (491–56857)	2623 (399–11241)	5287 (416–66330)
**SAT2 data sets**			**Substitution rates (s/bp/yr)**	**Substitution rates (s/bp/yr)**	**Substitution rates (s/bp/yr)**	***t***_**MRCA**_ **(y BP)**	***t***_**MRCA**_ **(y BP)**	***t***_**MRCA**_ **(y BP)**
** **	***n***	** Coverage**	**Constant**	**Exponential**	**Skyline**	**Constant**	**Exponential**	**Skyline**
SAT 2 combined	83	1948–2010	3.2 × 10^−3^ (2.0 × 10^−3^–4.4 × 10^−3^)	3.2 × 10^−3^ (2.1 × 10^−3^–4.4 × 10^−3^)	1.0 × 10^−3^ (0.7 × 10^−3^–1.4 × 10^−3^)	217 (108–366)	165 (114–230)	426 (300–584)
KENYA 2007 outbreak	11	2007	21.8 × 10^−3^ (2.1 × 10^−3^–46.9 × 10^−3^)	8.9 × 10^−3^ (0.1 × 10^−3^–34.4 × 10^−3^)	13.9 × 10^−3^ (0.01 × 10^−3^–31.7 × 10^−3^)	0.6 (0.3–2.0)	1.0 (0.3–6.2)	0.7 (0.3–4.7)
EGYPT 2012 outbreak	18	2012	34.7 × 10^−3^ (3.6 × 10^−3^–72.1 × 10^−3^)	33.3 × 10^−3^ (3.3 × 10^−3^–72.6 × 10^−3^)	23.1 × 10^−3^ (1.0 × 10^−3^–69.1 × 10^−3^)	0.3 (0.3–0.4)	0.3 (0.2–0.4)	0.3 (0.3–0.5)
ETHIOPIA 2009 outbreak	19	2009–2010	32.6 × 10^−3^ (10.5 × 10^−3^–60.2 × 10^−3^)	36.6 × 10^−3^ (7.6 × 10^−3^–164.4 × 10^−3^)	29.7 × 10^−3^ (2.7 × 10^−3^–74.7 × 10^−3^)	0.6 (0.6–0.7)	0.6 (0.6–0.7)	0.6 (0.6–0.7)
SOUTH AFRICA 2001 outbreak	11	2001	46.9 × 10^−3^ (10.1 × 10^−3^–84.9 × 10^−3^)	46.3 × 10^−3^ (1.1 × 10^−3^–81.0 × 10^−3^)	44.6 × 10^−3^ (13.3 × 10^−3^–83.7 × 10^−3^)	0.3 (0.3–0.5)	0.3 (0.3–0.5)	0.3 (0.3–0.4)
SAT 2 chronologically sampled	24	1948–2007	0.5 × 10^−3^ (0.004 × 10^−3^–1.0 × 10^−3^)	0.5 × 10^−3^ (0.01 × 10^−3^–1.0 × 10^−3^)	0.5 × 10^−3^ (0.1 × 10^−3^–1.0 × 10^−3^)	866 (289–3646)	799 (291–2279)	794 (323–2198)

The lower and upper 95% HPD intervals are given in parentheses. Rates and *t*_MRCA_ are given based on the models chosen in the Bayes Factor comparison.

The results for the SAT 2 serotype showed a similar pattern, where the SAT 2 combined data set exhibited higher median rates (3.2 (2.0–4.4), 3.2 (2.1–4.4) and 1.0 (0.7–1.4) × 10^−3^ s/nt/y) than the SAT 2 CHR data set (0.5 (0.004–1.0), 0.5 (0.01–1.0) and 0.5 (0.1–1.0) × 10^−3^ s/nt/y) for constant, exponential and skyline demographic models, respectively. As for SAT 1 the SAT 2 CLU data sets displayed higher median rates (here 18–90 times higher) compared to the SAT 2 CHR data set (Table 1).

The analyses of both serotypes further revealed that including sequences from CLUs in the data sets decreased the *t*_MRCA_, which is a natural consequence of the observed rate acceleration in CLUs. The combined SAT 1 data set including both CLU sequences had median depths for *t*_MRCA_ of 223 (121–395), 191 (127–278) and 386 (233–590) years before present (y BP), while the results for the CHR data set had median depths for *t*_MRCA_ of 4738 (491–56857), 2623 (399–11241) and 5287 (416–66330) y BP. SAT 2 data sets showed a similar difference, where the combined data set yielded median depths for *t*_MRCA_ of 217 (108–366), 165 (114–230) and 426 (300–584) y BP and the CHR median depths for *t*_MRCA_ of 866 (289–3646), 799 (291–2279) and 794 (323–2198) y BP (Table 1).

The choice of demographic model had an effect on the estimation of substitution rates. The exponential demographic model produced rates that were slightly higher than the substitution rate estimates using the constant and skyline demographic models when considering the combined and CHR data sets (Table 1). This underlines that the problem of rate inference cannot be considered separate from the problem of demographic inference. In the present study we treat demographic history as a ‘nuisance parameter’, and we also highlight that the data set–given its complex temporal and geographical structuring–is not suitable for demographic inference through the demographic models implemented in BEAST. Overall, however, this effect of the demographic prior did not change the relation between substitution rates and *t*_MRCA_ for the CHR and CLU data sets.

### Recombination and selection

We tested for the predominant type of selection acting on all codons within sequences and for evidence of recombination in the sequence data, as this is known to distort evolutionary analysis by overestimating substitution rate heterogeneity [35]. The SBP method found no statistical evidence for recombination within or between the two serotypes according to AIC and BIC scores; this result was confirmed by all methods in the RDP software. This corroborates earlier studies stressing that recombination is mainly constrained to non-structural proteins, with few observations in structural proteins [36]. We show results for *dN/dS* ratios in Table 2. All CLU data sets showed higher *dN/dS* ratios compared to the combined data set and the CHR data sets. We randomly subsampled the CHR data sets to match the sample sizes in the smallest of the corresponding CLU data sets; this was also done for the Kenya 2010 CLU, making the results from the selection analysis more comparable between sampling schemes. Randomly subsampled CHR data sets and CLU data sets showed considerable differences in *dN/dS* (Table 2).

**Table 2:** *dN/dS* ratios for all datasets.

**SAT1 data sets**			**Mean *dN/dS***
** **	***n***	** Coverage**	**SLAC**
SAT 1 combined	96	1937–2010	0.12 (0.11–0.13)
KENYA 2010 outbreak	56	2010	0.24 (0.16–0.33)
SAT 1 KENYA random 1	12	2010	0.40 (0.14–0.85)
SAT 1 KENYA random 2	12	2010	0.18 (0.09–0.32)
SAT 1 KENYA random 3	12	2010	0.29 (0.15–0.51)
SAT 1 KENYA random 4	12	2010	0.30 (0.15–0.51)
SAT 1 KENYA random 5	12	2010	0.23 (0.09–0.47)
NIGER/NIGERIA outbreak	12	1975–1977	0.18 (0.10–0.31)
SAT 1 random 1	12	1948–1999	0.12 (0.11–0.14)
SAT 1 random 2	12	1937–2007	0.10 (0.09–0.12)
SAT 1 random 3	12	1949–2006	0.11 (0.10–0.13)
SAT 1 random 4	12	1949–2003	0.11 (0.09–0.13)
SAT 1 random 5	12	1976–2006	0.11 (0.09–0.12)
SAT 1 chronologically sampled	28	1937–2008	0.12 (0.11–0.13)
**SAT2 data sets**			**Mean *dN/dS***
** **	***n***	** Coverage**	**SLAC**
SAT 2 combined	83	1948–2010	0.09 (0.09–0.10)
KENYA 2007 outbreak	11	2007	0.21 (0.11–0.35)
EGYPT 2012 outbreak	18	2012	0.18 (0.04–0.46)
ETHIOPIA 2009 outbreak	19	2009–2010	0.15 (0.05–0.35)
SOUTH AFRICA 2001 outbreak	11	2001	0.33 (0.12–0.71)
SAT 2 random 1	11	1948–2000	0.07 (0.06–0.08)
SAT 2 random 2	11	1948–2007	0.09 (0.08–0.10)
SAT 2 random 3	11	1981–2007	0.09 (0.08–0.11)
SAT 2 random 4	11	1948–2007	0.08 (0.08–0.10)
SAT 2 random 5	11	1948–2004	0.10 (0.08–0.12)
SAT 2 chronologically sampled	24	1948–2007	0.09 (0.08–0.10)

Single likelihood ancestor counting (SLAC) P < 0.1. Serotype sequences constituted 221 amino acids (SAT 1), 216 amino acids (SAT 2).

## Discussion

### Phylogenetic Analysis

This study demonstrates an overlooked problem in RNA virus sequence analysis. Duchêne *et al*. [16] recently investigated a similar issue across major groups of viruses and found declining substitution rates over time both within and between diverse groups of viruses. In this study, we found that substitution rates and the most recent common ancestor inferred for FMDV can be biased by the inclusion of temporally clustered sequences due to the effect of confounding mutation rates with longer-term substitution rates. This phenomenon has also been referred to as time-dependent rate variation [37]. These results have important implications. Specifically, we advocate avoiding using densely sampled sequences which temporally cover one to a few years when the long-term substitution rates is the subject of interest.

### Selection pattern

All clustered data sets showed considerably higher *dN/dS* values compared to both the combined and the evenly sampled data sets respectively (see Table 2). This suggests that there are fewer codons experiencing purifying selection for the clustered data sets and reveal putative different molecular evolutionary patterns over different time scales in FMDV, confirming the distinction between a short-term mutation rate (where selection has not had time to work) and a longer-term substitution rate (where selection is more evident). The different number of codons under selection in the clustered data sets will affect the substitution rates differently. Whereas the clustered data sets–having higher *dN/dS* ratios–will have substitution rates closer to the mutation rate, samples with larger temporal spacing will show the effects of purifying selection and be closer to the true long-term substitution rate. This observation emphasizes the need for further studies to test the impact of variable selection pressures within and between outbreaks.

### Date permutation and sampling considerations

The results from the date permutation test revealed discrepancies between serotypes. Whereas support for a sufficient temporal signal was absent from half of the SAT 1 data sets, we saw strong support for the temporal signal in the SAT 2 serotype data sets using the exponential demographic model. Our approach for this test followed that of Ramsden *et al*. [27], where all tip-dates are included in the permutation. However, later studies [38,39] proposed an improvement to this method by performing ‘clustered permutation’, where sequences are divided into groups according to their sampling time. This was not feasible in our study due to the way CLU and CHR data sets were divided (see Definition of temporal sample clusters). Furthermore, our procedure for selecting sequences to be put in to the CHR data sets may cause a bias in the estimated *t*_MRCA_, as choosing samples from different geographical areas and topotypes naturally causes longer coalescence times. For example, the SAT 1 CHR data set, using the exponential demographic model, had very long terminal branches (see Fig 1), which means that tip-date permutation (shuffling tip dates within a 73 year time span) has a relatively minor effect. In this case there will be little difference between the real and the date-permuted rates, suggesting a lack of temporal signal. Instead the effect is probably more due to the effect of the demographic prior (exponential demographic model) which in this case appears to dominate the temporal signal of the samples. However, a previous study has shown that high temporal information content tends to overcome the demographic prior under different sampling regimes resembling ours [40].

Important decisions about the composition of sequences must be made to avoid biases in estimates of *t*_MRCA_ and substitution rates in FMDV analyses. As mentioned, one part of this issue could be resolved by avoiding too dense temporal sampling in analyses of long-term evolutionary rates. Another issue involves geographical structure [3,41,42], which we did not investigate here. Uncritical sampling from a structured population, while assuming panmixia, could lead to spurious demographic signals with a strong recent decline in effective population size and hence result in a biased estimate of the *t*_MRCA_ [43].

## Conclusion

This study emphasizes the sensitivity of evolutionary analyses to the temporal sampling structure in FMDV. This is evident from considerable differences in substitution rates and *t*_MRCA_ as well as different levels of selection between temporally clustered and temporally dispersed data sets. Based on our results, we suggest that careful consideration of the sampling scheme is needed to assess the unbiased long-term evolutionary parameters within FMDV and other RNA viruses.

## Supporting Information

S1 Appendix Sequence accession numbers and topotype for FMDV isolates (XLSX). (XLSX)

S2 Appendix Input files for BEAST (XML). (ZIP)

S3 Appendix Datasets (Nexus). (ZIP)

S1 Fig Date permutation plots (pdf). (PDF)

S2 Fig Date permutation plots (pdf). (PDF)

S3 Fig Date permutation plots (pdf). (PDF)

S4 Fig Date permutation plots (pdf). (PDF)

S5 Fig Date permutation plots (pdf). (PDF)

S6 Fig Date permutation plots (pdf). (PDF)

S7 Fig Date permutation plots (pdf). (PDF)

S8 Fig Date permutation plots (pdf). (PDF)

S9 Fig Date permutation plots (pdf). (PDF)

S10 Fig Date permutation plots (pdf). (PDF)

S11 Fig Date permutation plots (pdf). (PDF)

S12 Fig Date permutation plots (pdf). (PDF)

S13 Fig Date permutation plots (pdf). (PDF)

S14 Fig Date permutation plots (pdf). (PDF)

S15 Fig Date permutation plots (pdf). (PDF)

S16 Fig Date permutation plots (pdf). (PDF)

S17 Fig Date permutation plots (pdf). (PDF)

S18 Fig Date permutation plots (pdf). (PDF)

S19 Fig Date permutation plots (pdf). (PDF)

S20 Fig Date permutation plots (pdf). (PDF)
