# Supplementary figures and images for: Time Clustered Sampling Can Inflate the Inferred Substitution Rate in Foot-And-Mouth Disease Virus Analyses
Source: PLoS One. 2015 Dec 2;10(12):e0143605. doi: 10.1371/journal.pone.0143605 (PMC4667911; doi:10.1371/journal.pone.0143605)

# Date-Randomization for sat1CHR

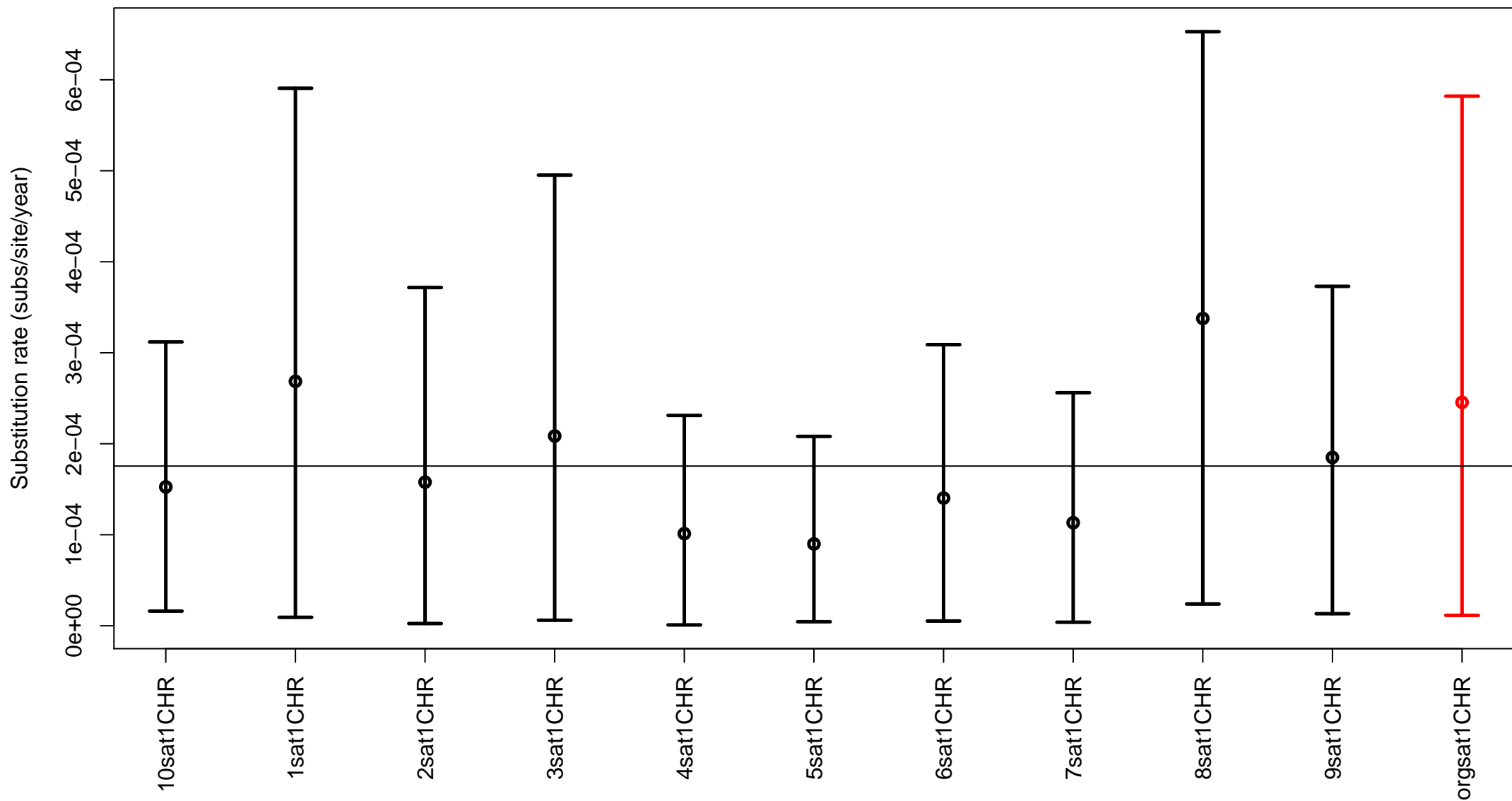

Supplement: S1 Fig — (PDF) [file pone.0143605.s004.pdf]

# Date-Randomization for sat1COMB

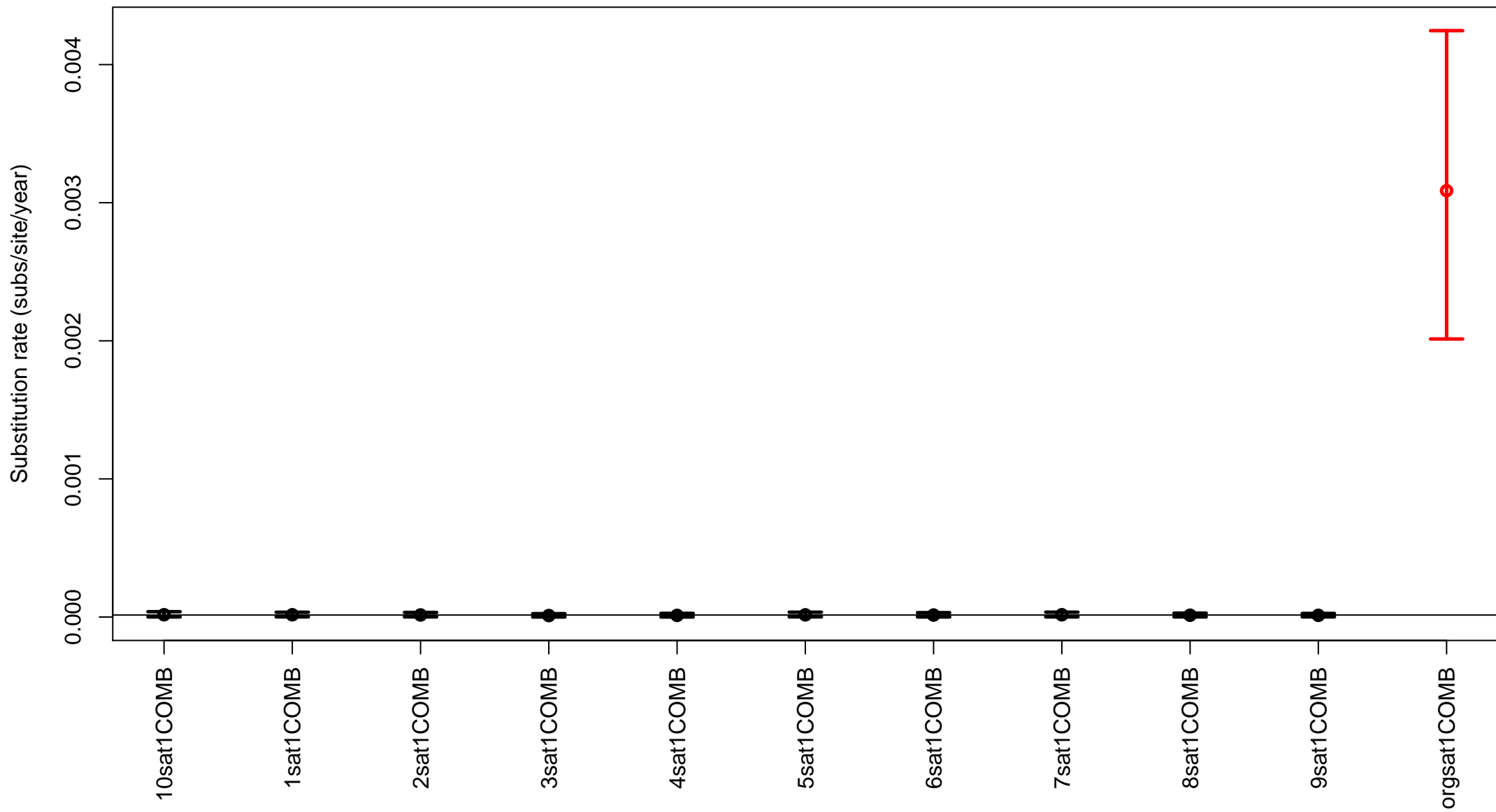

Supplement: S2 Fig — (PDF) [file pone.0143605.s005.pdf]

# Date-Randomization for sat1KENYA

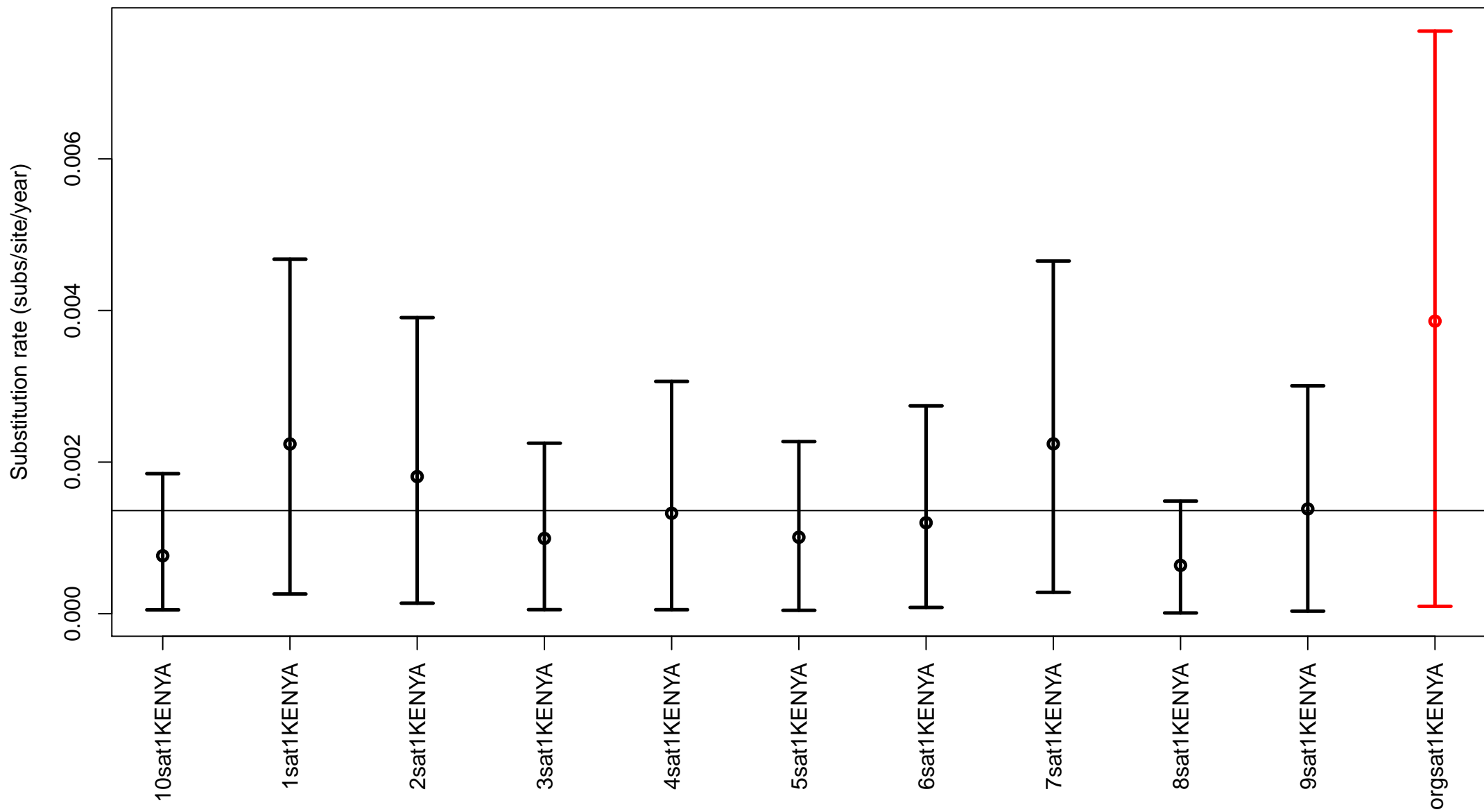

Supplement: S3 Fig — (PDF) [file pone.0143605.s006.pdf]

Date-Randomization for sat1NigExp

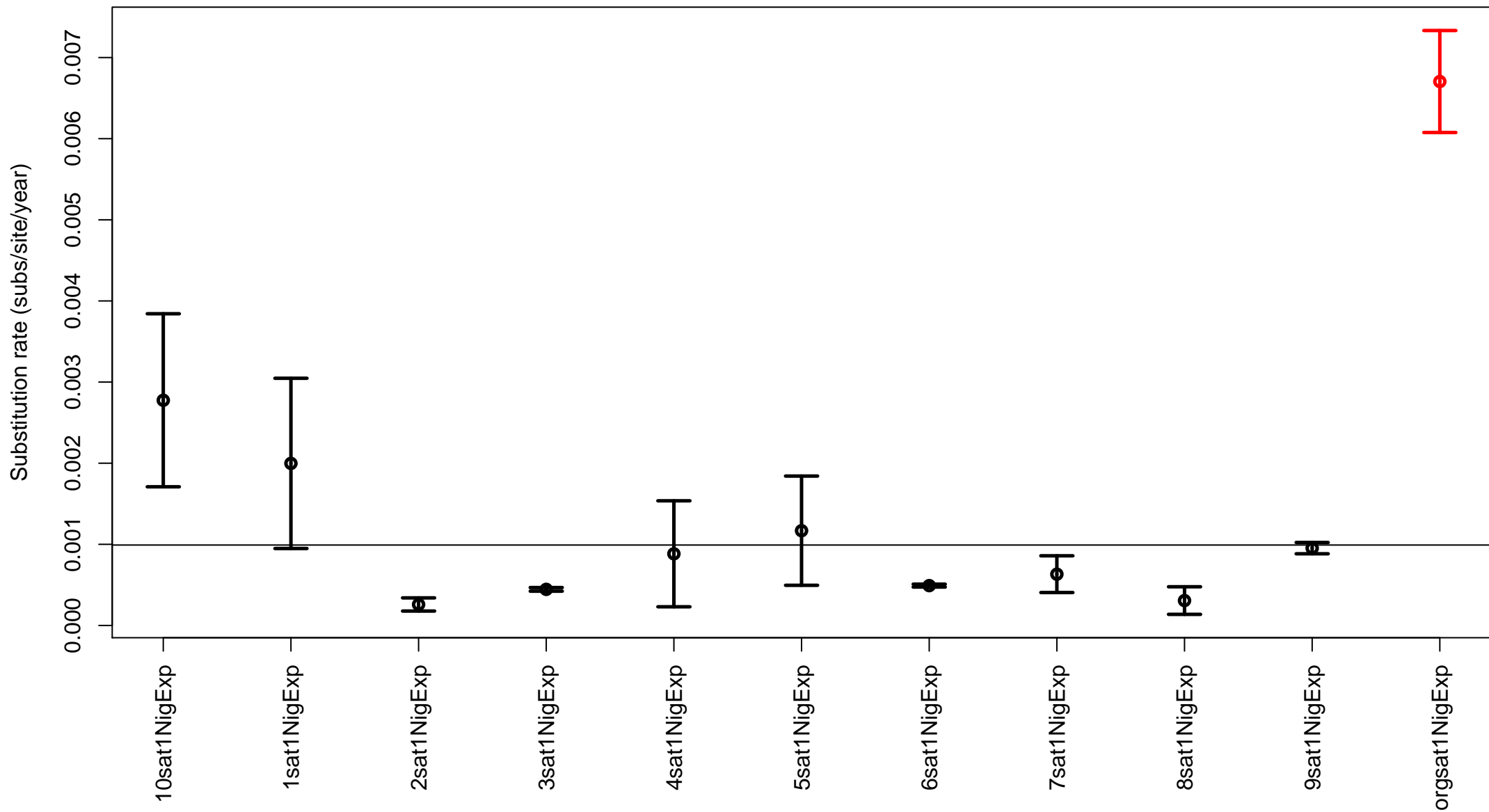

Supplement: S4 Fig — (PDF) [file pone.0143605.s007.pdf]

# Date-Randomization for sat2CHR

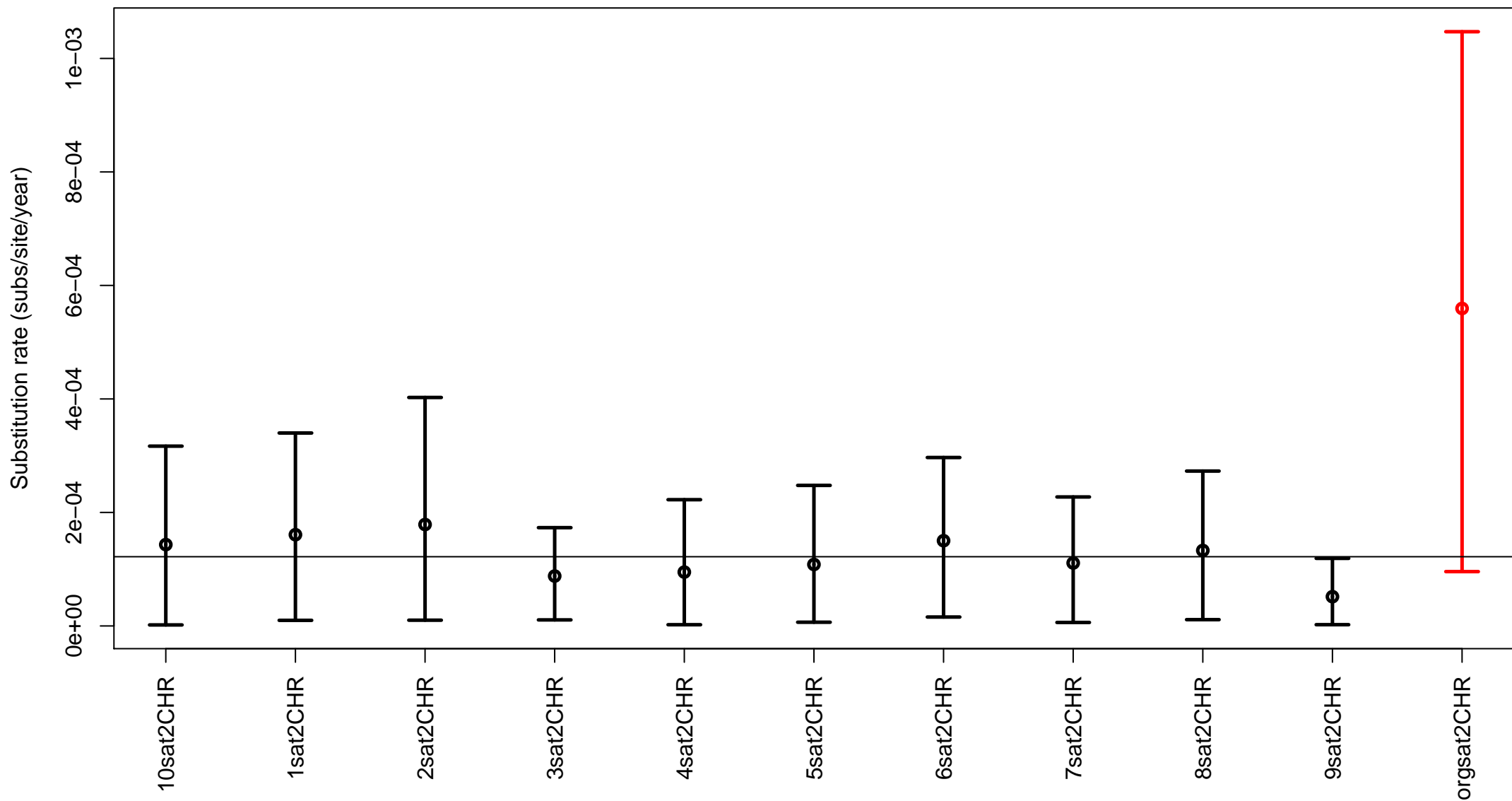

Supplement: S5 Fig — (PDF) [file pone.0143605.s008.pdf]

# Date-Randomization for sat2COMB

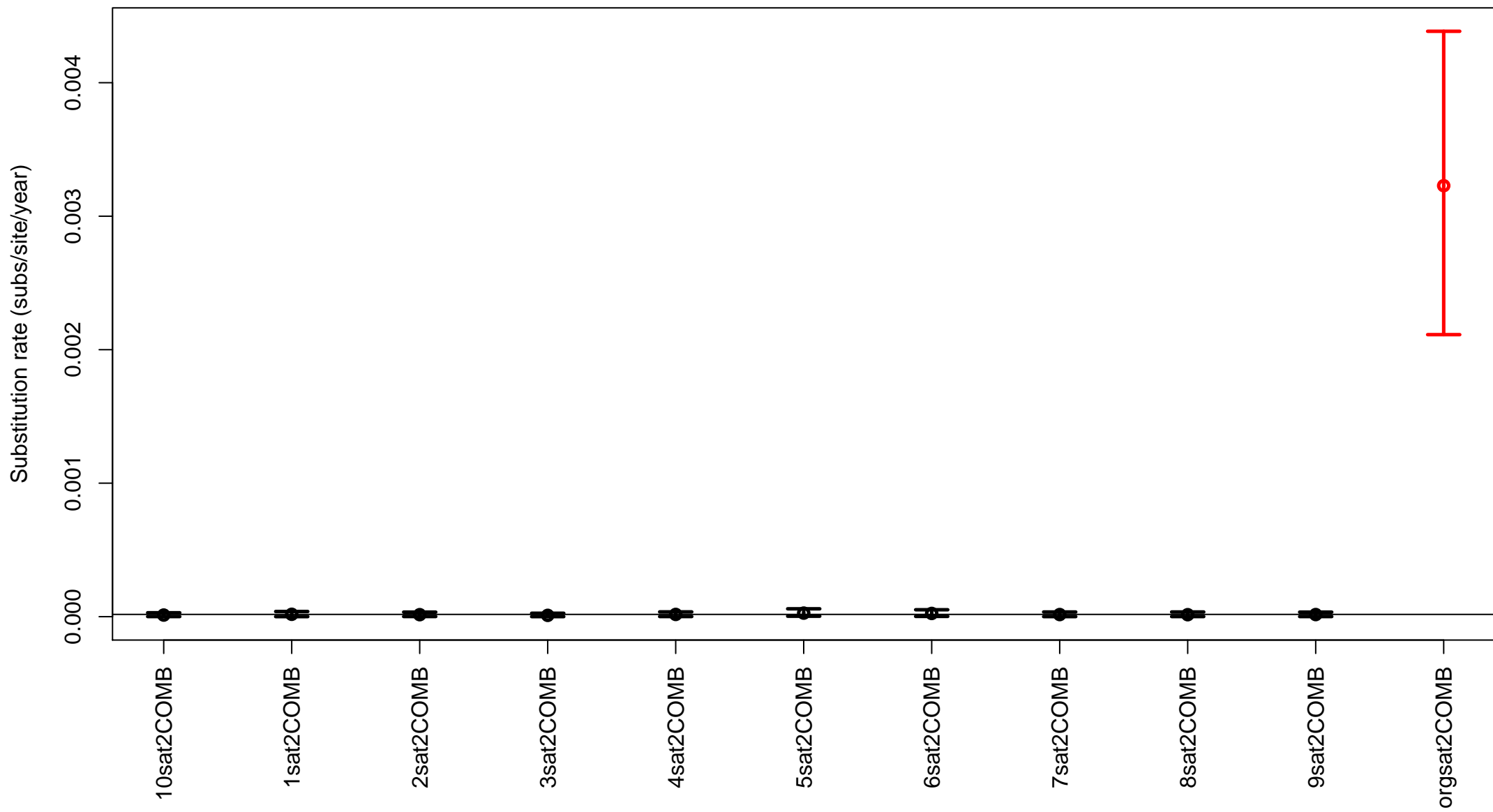

Supplement: S6 Fig — (PDF) [file pone.0143605.s009.pdf]

Date-Randomization for sat2EgyExp

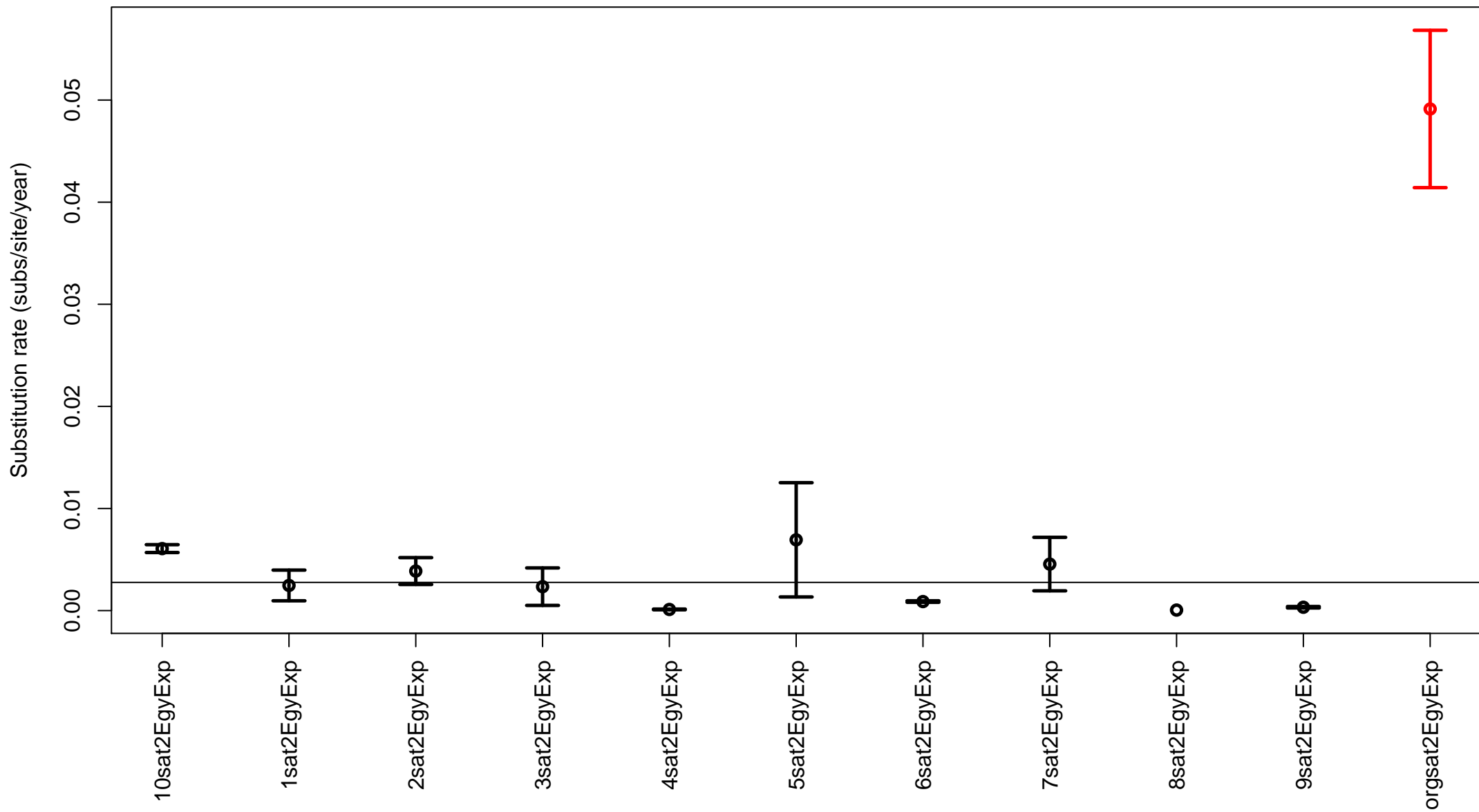

Supplement: S7 Fig — (PDF) [file pone.0143605.s010.pdf]

Date-Randomization for sat2EthExp

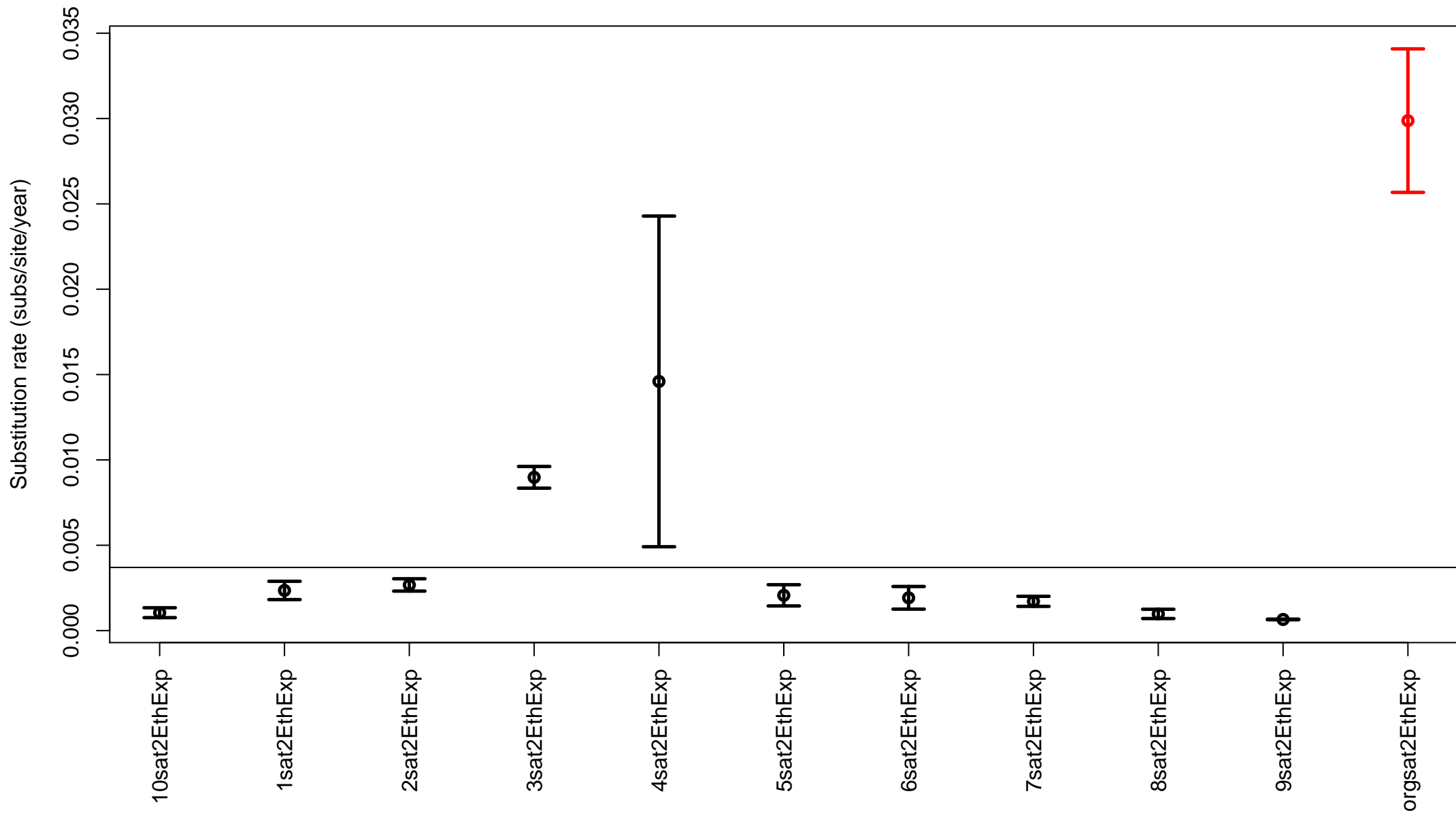

Supplement: S8 Fig — (PDF) [file pone.0143605.s011.pdf]

# Date-Randomization for sat2KenExp

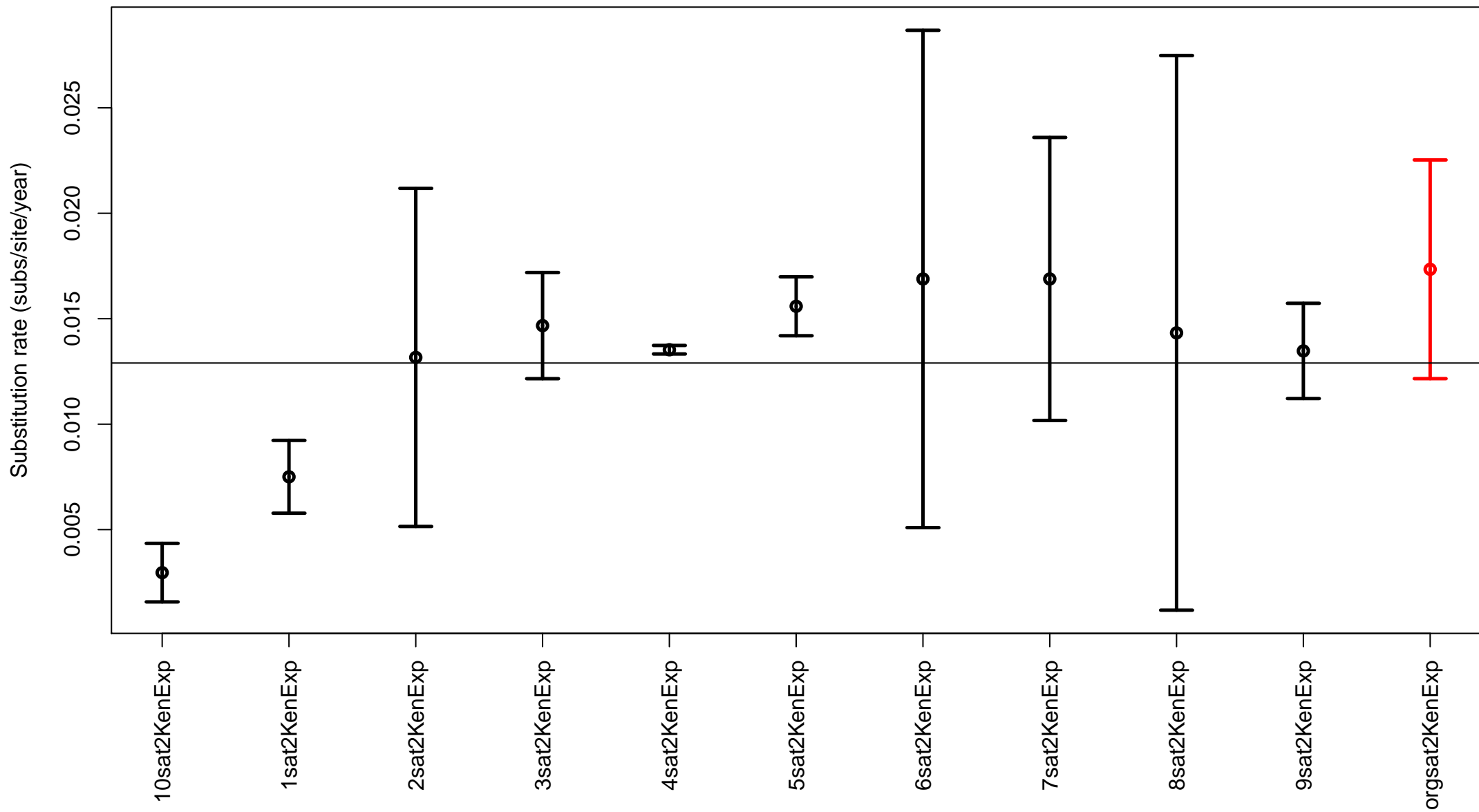

Supplement: S9 Fig — (PDF) [file pone.0143605.s012.pdf]

# Date-Randomization for sat2SouExp

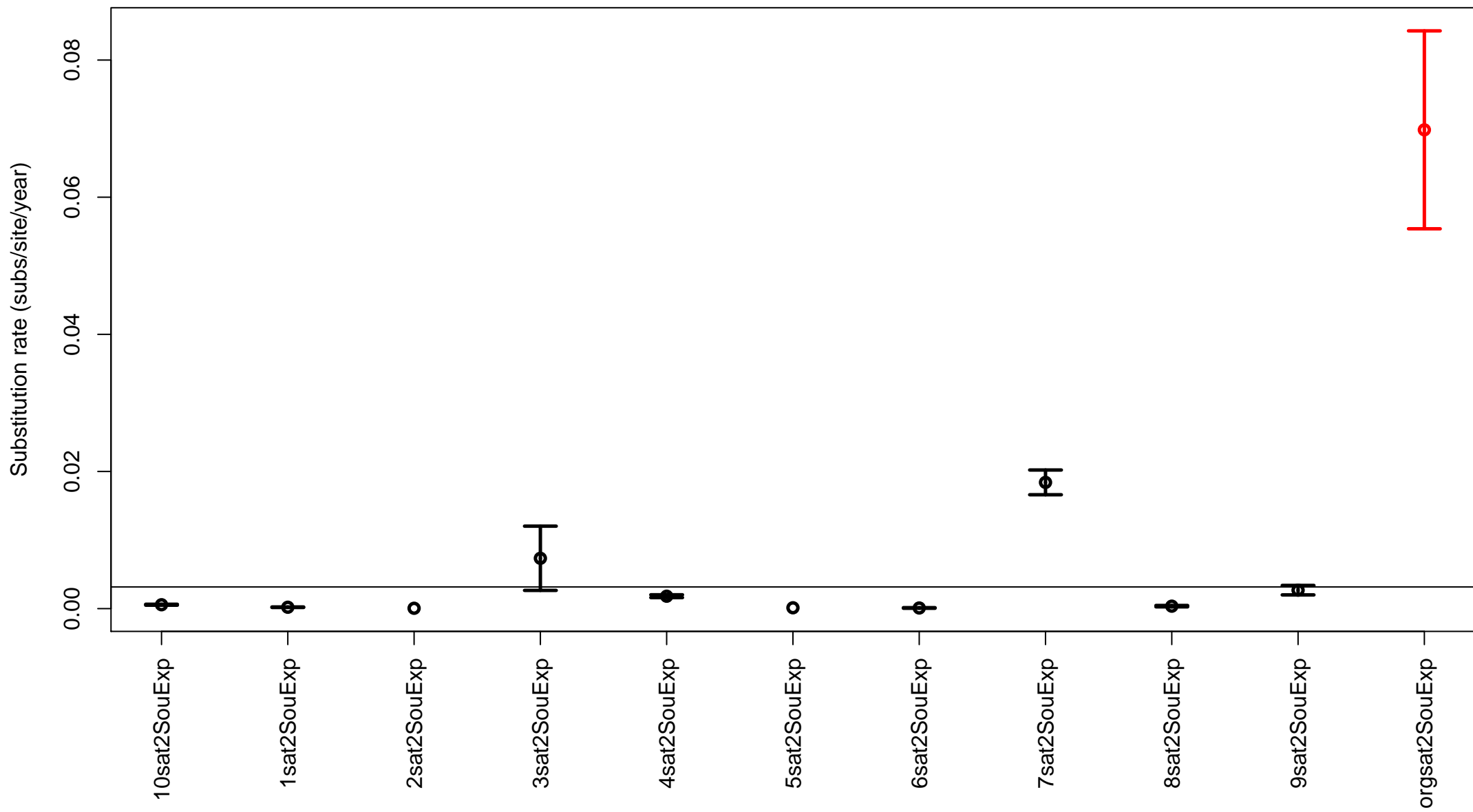

Supplement: S10 Fig — (PDF) [file pone.0143605.s013.pdf]

# Date-Randomization for sat1CHR

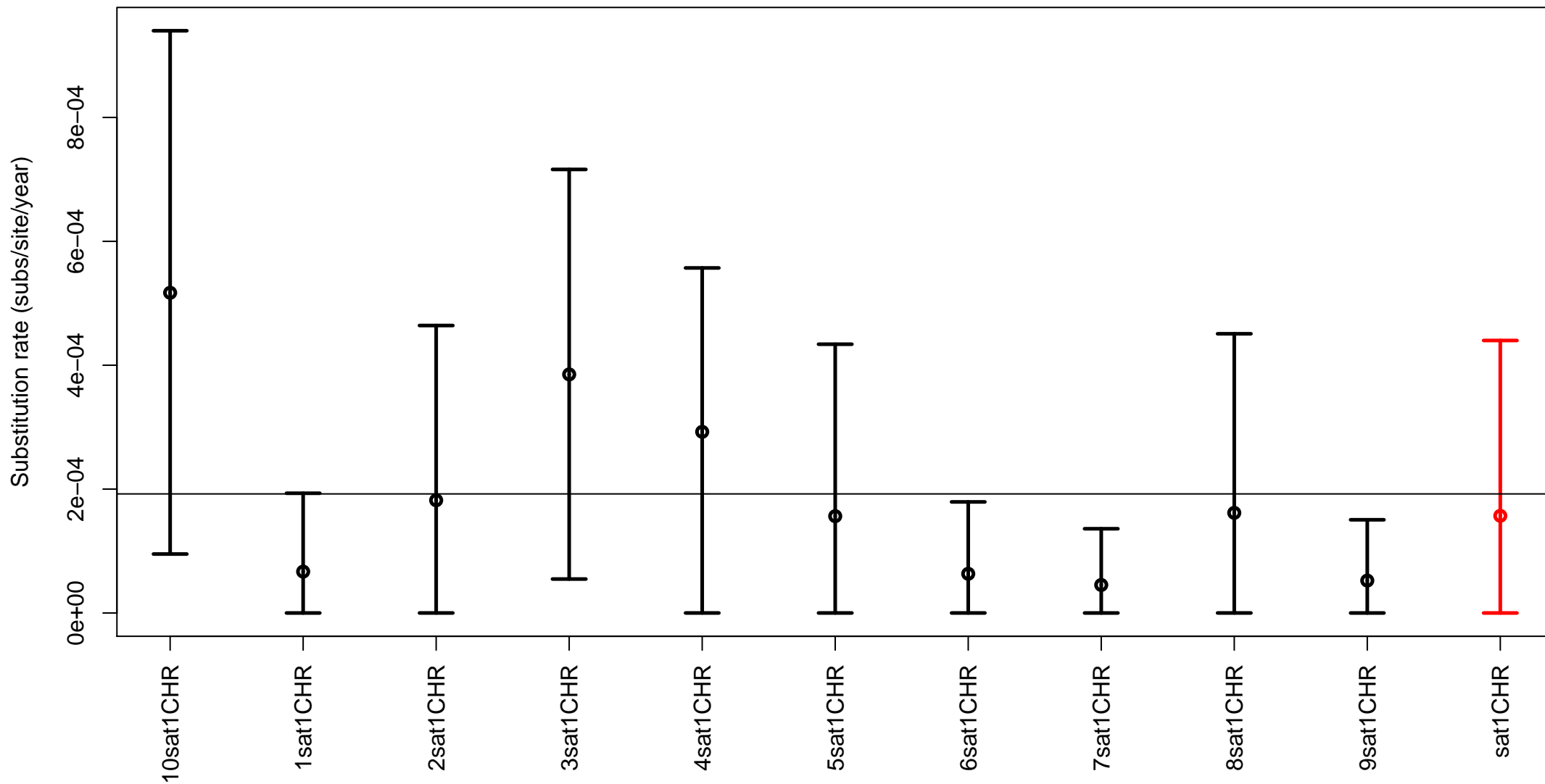

Supplement: S11 Fig — (PDF) [file pone.0143605.s014.pdf]

# Date-Randomization for sat1COMB

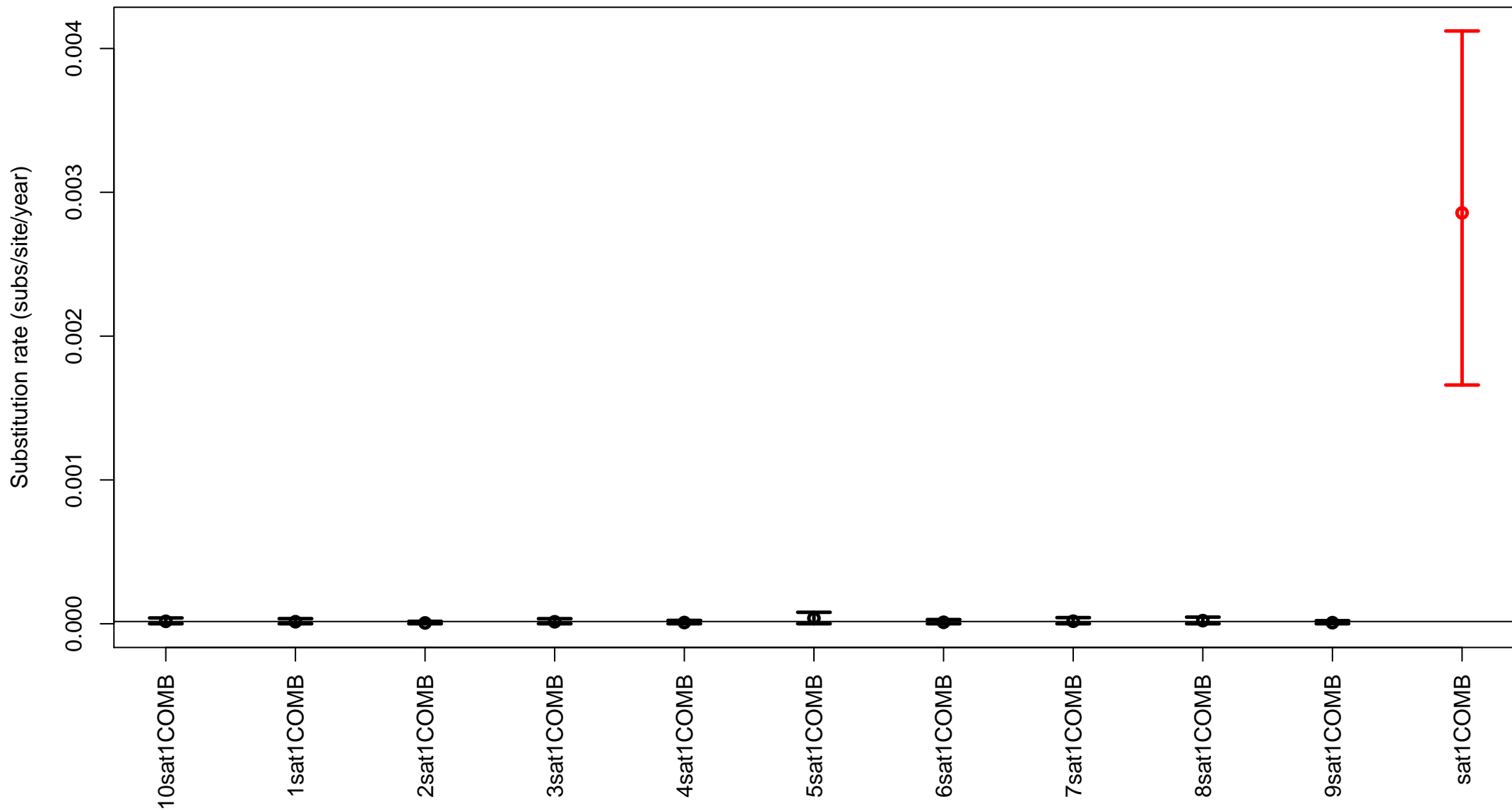

Supplement: S12 Fig — (PDF) [file pone.0143605.s015.pdf]

# Date-Randomization for NIGERIA\_7576

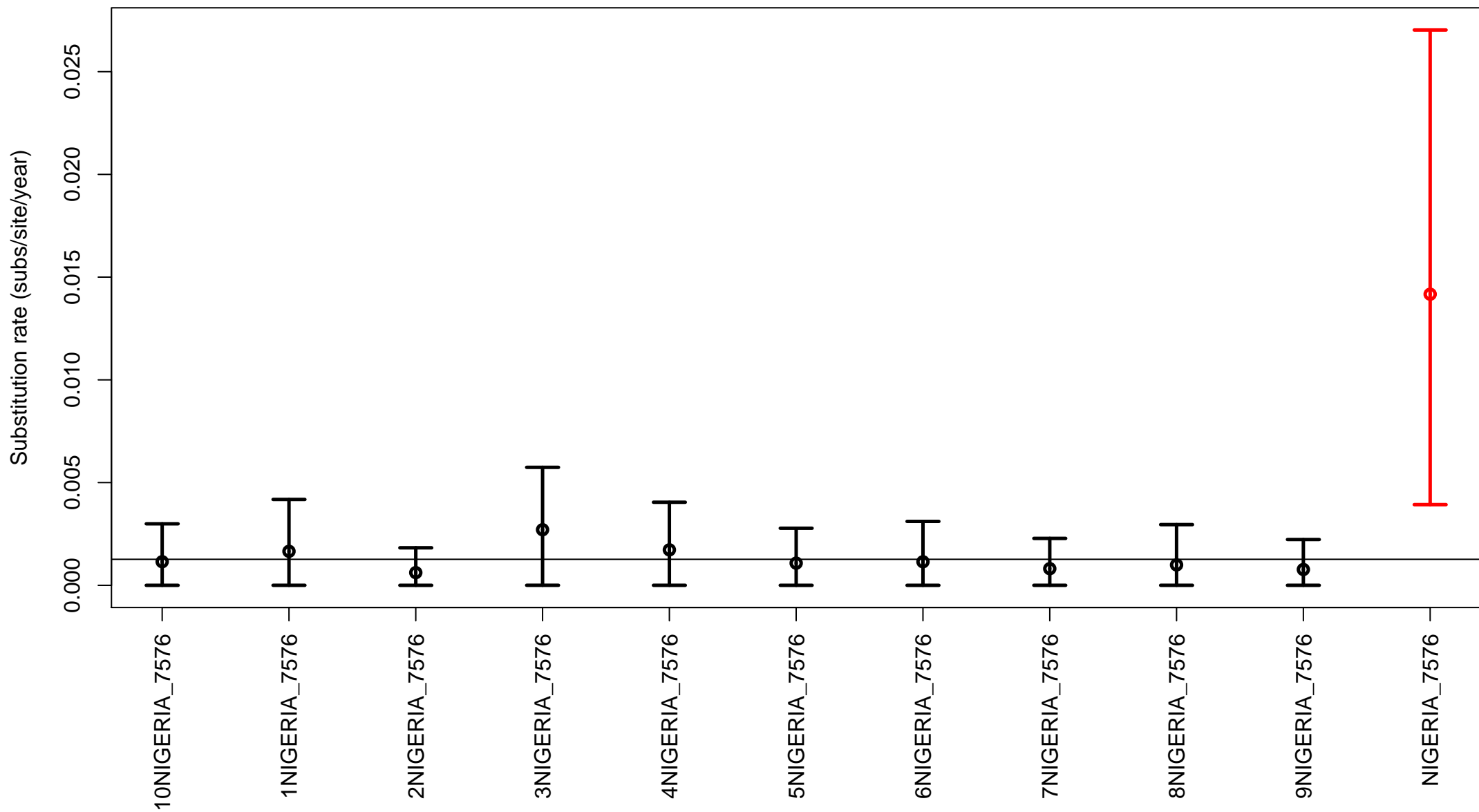

Supplement: S13 Fig — (PDF) [file pone.0143605.s016.pdf]

# Date-Randomization for sat1KENYA

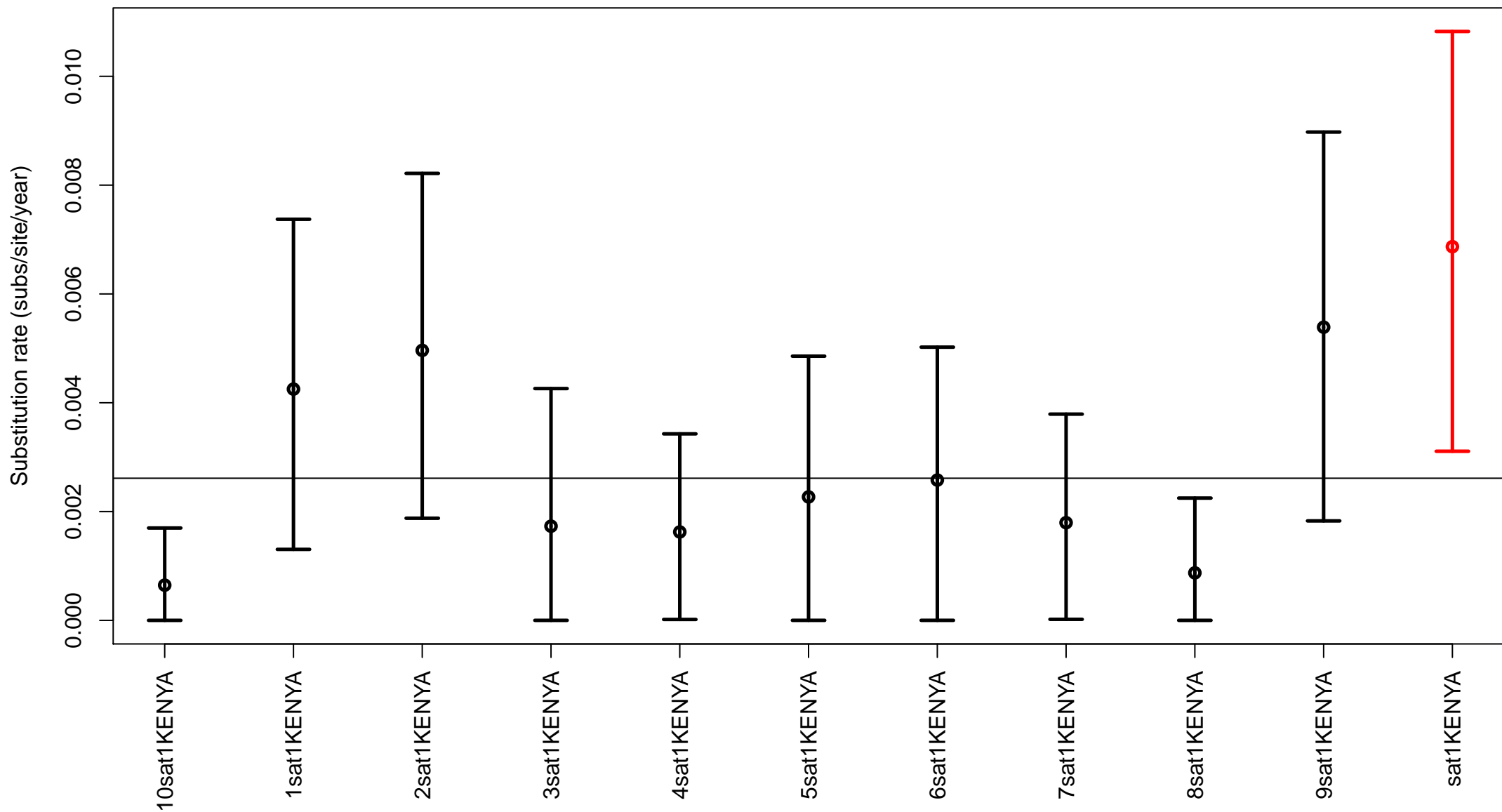

Supplement: S14 Fig — (PDF) [file pone.0143605.s017.pdf]

# Date-Randomization for sat2CHR

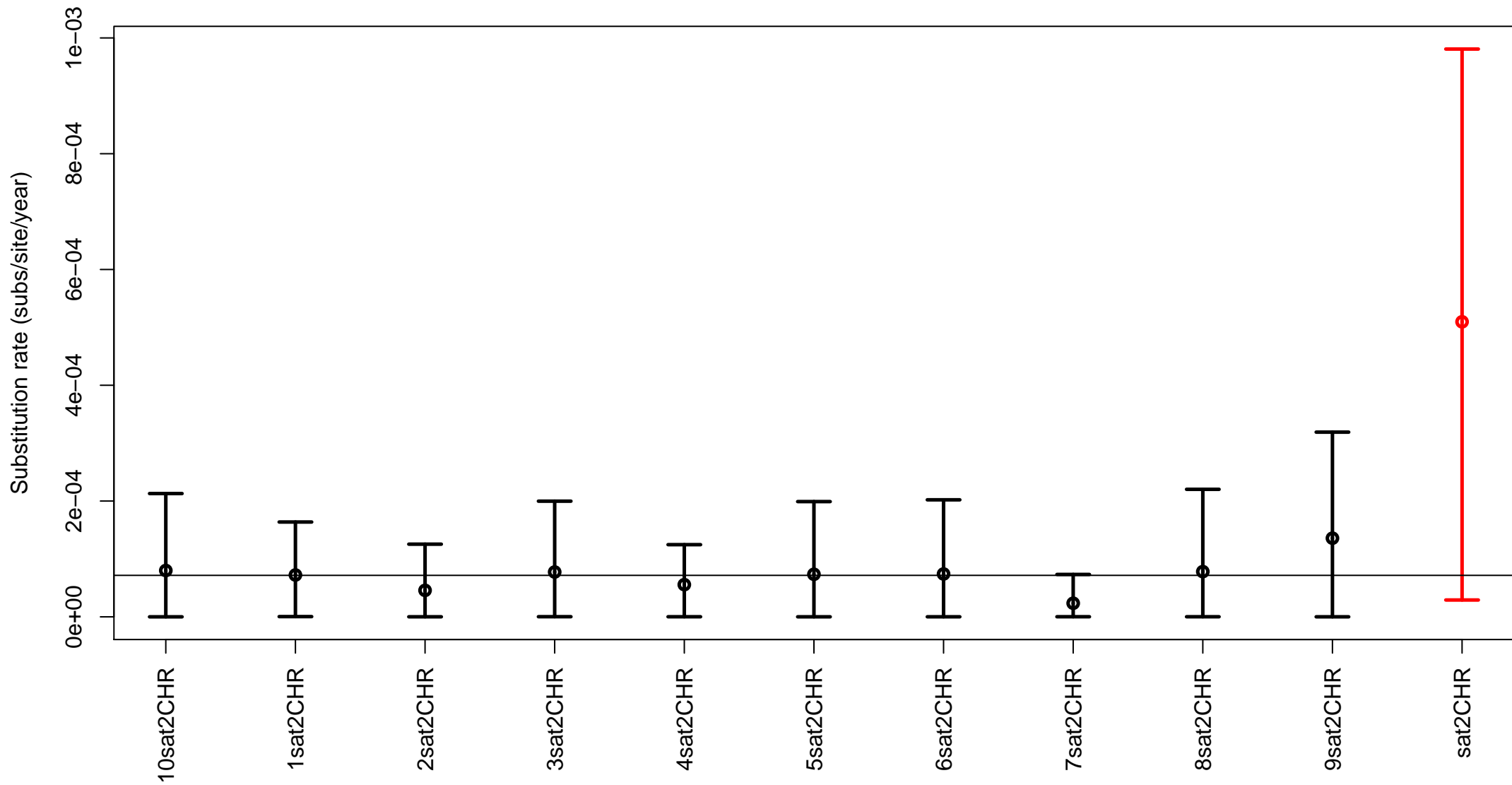

Supplement: S15 Fig — (PDF) [file pone.0143605.s018.pdf]

# Date-Randomization for sat2COMB

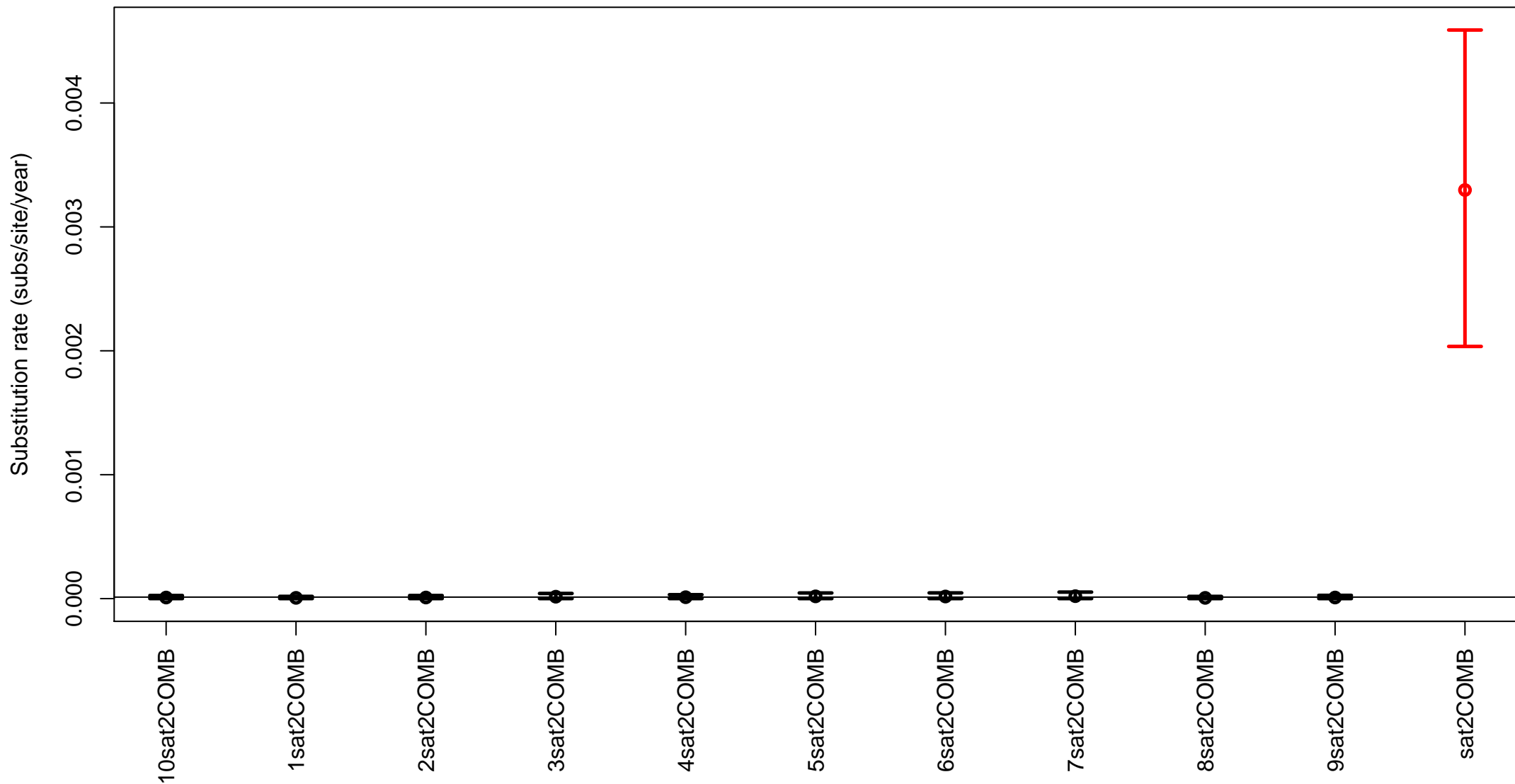

Supplement: S16 Fig — (PDF) [file pone.0143605.s019.pdf]

# Date-Randomization for Egypt\_2012

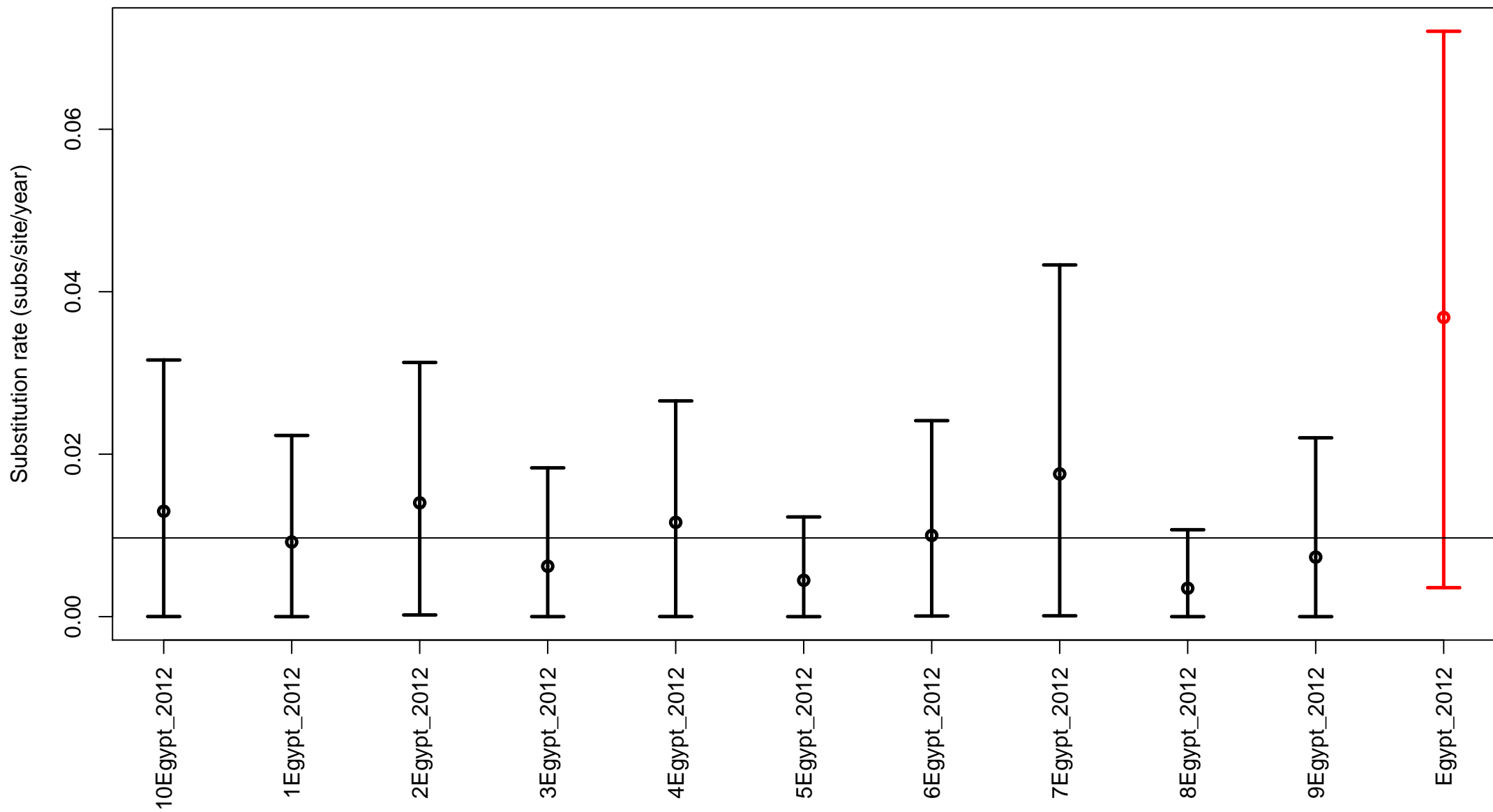

Supplement: S17 Fig — (PDF) [file pone.0143605.s020.pdf]

# Date-Randomization for KENYA\_2007

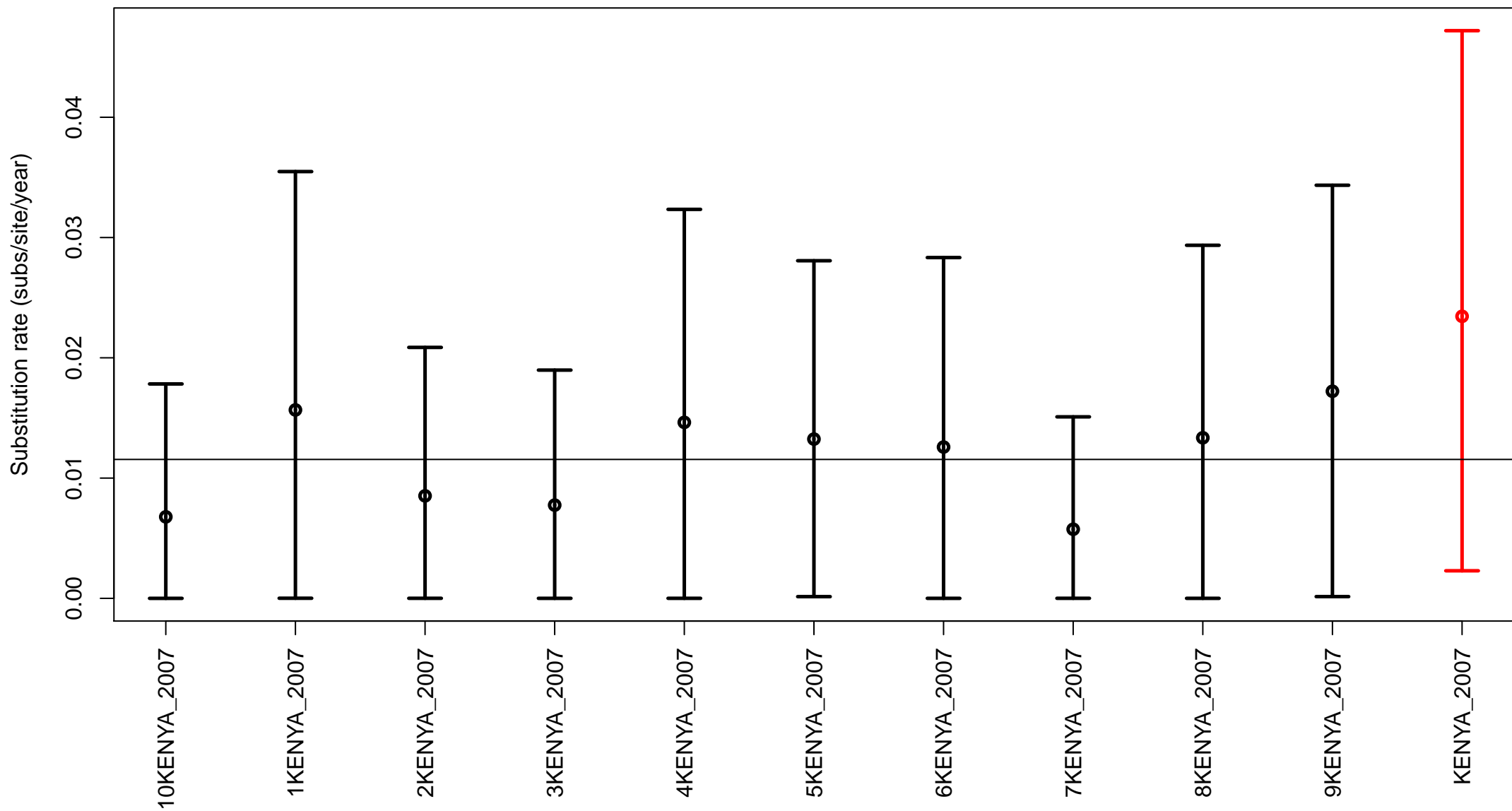

Supplement: S18 Fig — (PDF) [file pone.0143605.s021.pdf]

Date-Randomization for Ethiopia\_2009

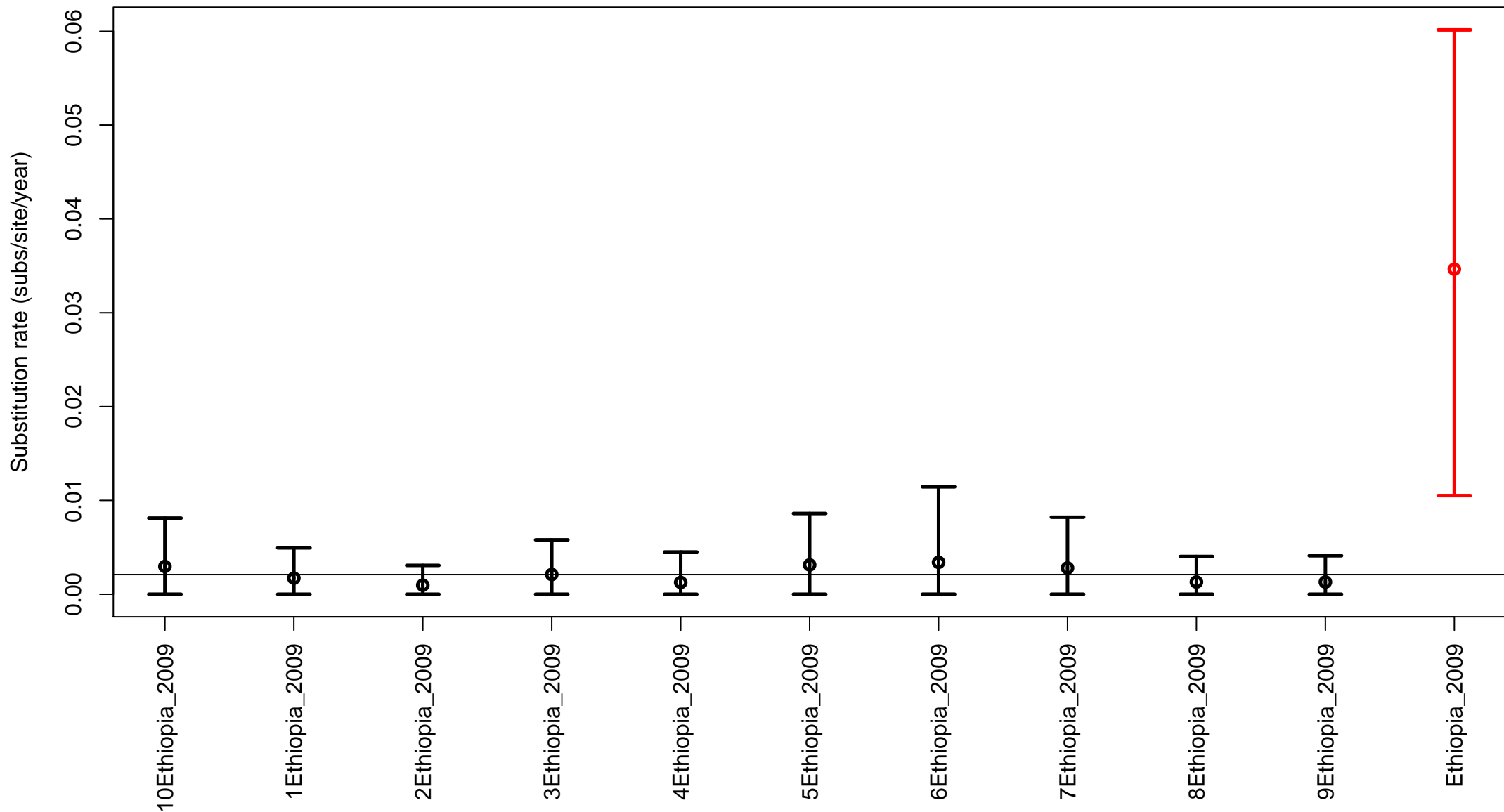

Supplement: S19 Fig — (PDF) [file pone.0143605.s022.pdf]

# Date-Randomization for South\_Africa

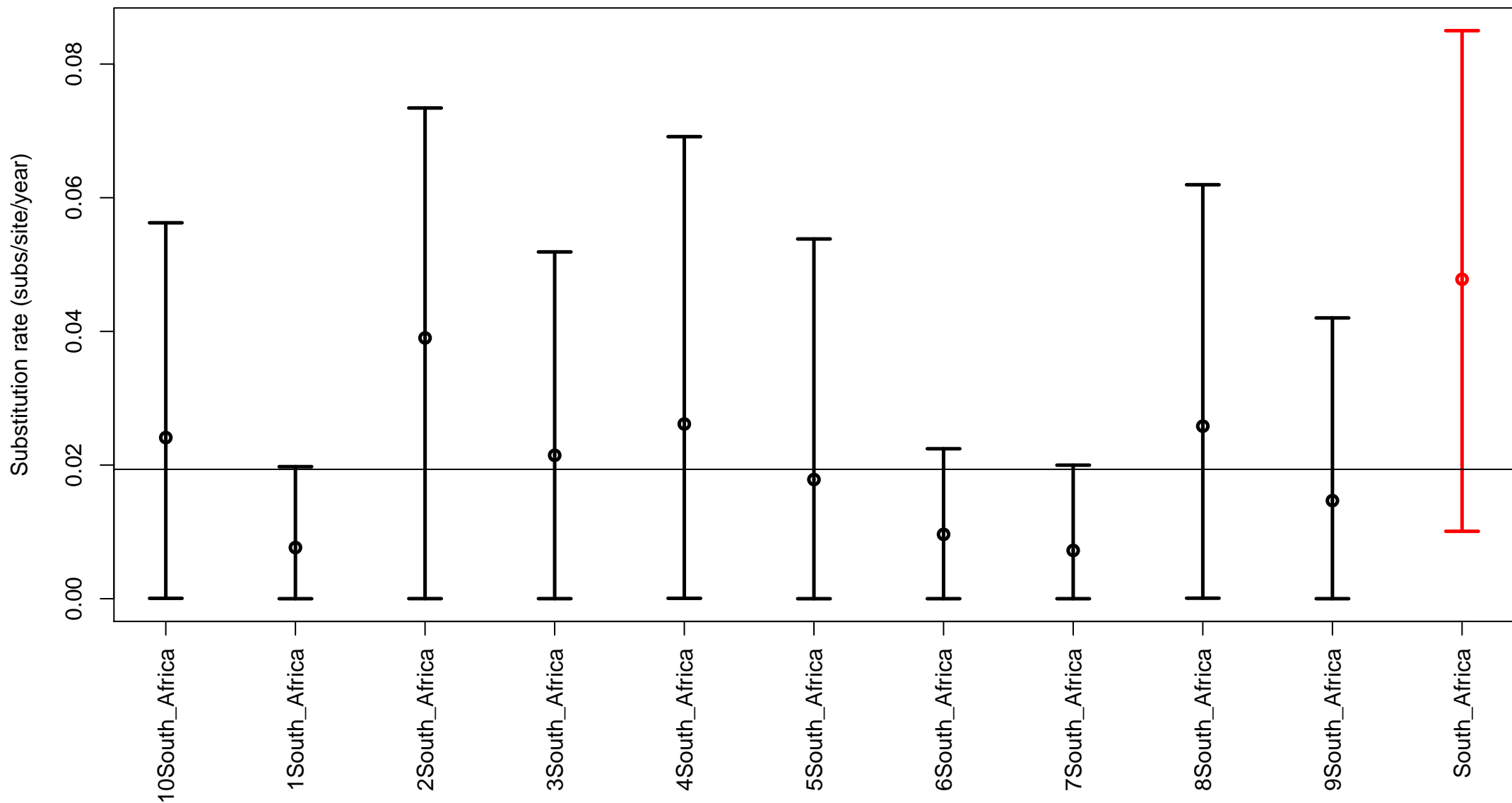

Supplement: S20 Fig — (PDF) [file pone.0143605.s023.pdf]
